# A genome-scale metabolic reconstruction of *Pseudomonas putida *KT2440: *i*JN746 as a cell factory

**DOI:** 10.1186/1752-0509-2-79

**Published:** 2008-09-16

**Authors:** Juan Nogales, Bernhard Ø Palsson, Ines Thiele

**Affiliations:** 1 Departamento de Microbiología Molecular, Centro de Investigaciones Biológicas-CSIC, Ramiro de Maeztu 9, Madrid, 28040, Spain; 2 Department of Bioengineering, University of California, San Diego, Gilman Drive 9500, 92093 La Jolla, CA, USA; 3 PhD program in Bioinformatics, University of California, San Diego, Gilman Drive 9500, 92093 La Jolla, CA, USA

## Abstract

**Background:**

*Pseudomonas putida *is the best studied pollutant degradative bacteria and is harnessed by industrial biotechnology to synthesize fine chemicals. Since the publication of *P. putida *KT2440's genome, some *in silico *analyses of its metabolic and biotechnology capacities have been published. However, global understanding of the capabilities of *P. putida *KT2440 requires the construction of a metabolic model that enables the integration of classical experimental data along with genomic and high-throughput data. The constraint-based reconstruction and analysis (COBRA) approach has been successfully used to build and analyze *in silico *genome-scale metabolic reconstructions.

**Results:**

We present a genome-scale reconstruction of *P. putida *KT2440's metabolism, *i*JN746, which was constructed based on genomic, biochemical, and physiological information. This manually-curated reconstruction accounts for 746 genes, 950 reactions, and 911 metabolites. *i*JN746 captures biotechnologically relevant pathways, including polyhydroxyalkanoate synthesis and catabolic pathways of aromatic compounds (e.g., toluene, benzoate, phenylacetate, nicotinate), not described in other metabolic reconstructions or biochemical databases. The predictive potential of *i*JN746 was validated using experimental data including growth performance and gene deletion studies. Furthermore, *in silico *growth on toluene was found to be oxygen-limited, suggesting the existence of oxygen-efficient pathways not yet annotated in *P. putida*'s genome. Moreover, we evaluated the production efficiency of polyhydroxyalkanoates from various carbon sources and found fatty acids as the most prominent candidates, as expected.

**Conclusion:**

Here we presented the first genome-scale reconstruction of *P. putida*, a biotechnologically interesting all-surrounder. Taken together, this work illustrates the utility of *i*JN746 as i) a knowledge-base, ii) a discovery tool, and iii) an engineering platform to explore *P. putida*'s potential in bioremediation and bioplastic production.

## Background

*Pseudomonas putida *is a non-pathogenic member of rRNA group I of the genus *Pseudomonas *that colonizes many different environments and is well known for its broad metabolic versatility and genetic plasticity [1,2]. *P. putida *KT2440 is a TOL plasmid cured, spontaneous restriction deficient derivative of *P. putida *mt-2 [3,4]. This strain represents the first host-vector biosafety system for cloning in gram-negative soil bacteria and hence, has been extensively used as a host for gene cloning and expression of heterologous genes [5-8]. Consequently, large efforts have been made in exploiting these capacities in a diverse range of biotechnological applications including i) bioremediation of contaminated areas [9,10]; ii) quality improvement of fossil fuels, e.g., by desulphurization [11]; iii) biocatalytic production of fine chemicals [9,12-14]; iv) production of bioplastic [15-17]; and v) as agents of plant growth promotion and plant pest control [18,19].

Since the publication of *P. putida *KT2440's genome [20], our knowledge about this strain has significantly increased [21] and various "-omics" data sets have become available, such as transcriptomic [22,23], proteomic [24], and fluxomic data [25,26]. Subsequently, some *in silico *analyses of its metabolic and biotechnological capacities have been published [27,28]. However, systemic understanding of metabolic and biotechnology capabilities of *P. putida *KT2440 requires the construction of a more comprehensive model enabling the integration of the canonical experimental data along with genomic and high-throughput data in a hierarchical and coherent fashion [29].

The constraint-based reconstruction and analysis (COBRA) approach is one possible modeling approach that uses stoichiometric information about biochemical transformation taking place in a target organism to construct the model. While a metabolic reconstruction is unique to the target organism one can derive many different condition-specific models from a single reconstruction. This conversion of a metabolic reconstruction of an organism into models requires the imposition of physicochemical and environmental constraints to define systems boundaries [30-32]. The conversion also includes the transformation of the reaction list into a computable, mathematical matrix format. In this so-called S matrix, where S stands for stoichiometric, the rows correspond to the network metabolites and the columns to the network reactions. The coefficients of the substrates and products of each reaction are entered in the corresponding cell of the matrix. This conversion can be done automatically (e.g., using the Matlab-based COBRA toolbox [33]). Once in this format, numerous mathematical tools can be used to interrogate the metabolic network properties *in silico*. Many of the published mathematical tools have been reviewed [34] and encoded in Matlab format [33]. A large subset of these tools relies on linear programming (LP), a mathematical tool used to find a solution to an optimization problem (e.g., maximal possible growth rate of my metabolic network under a given set of environmental constraints). While LP-based tools are very helpful in studying reconstructed metabolic networks, some questions may better be addressed without having to choose an objective function. Those methods are called unbiased methods, in contrast to biased LP-based methods, because they identify all feasible flux distributions under the given set of environmental constraints rather than only the optimal distributions. The COBRA approach [30,32] has been successfully used to build and analyze genome-scale *in silico *reconstructions for representatives of archaea (e.g.,*Methanosarcina barkeri *[35]), of bacteria (e.g., *E. coli *[36]; *B. subtilis *[37]; *H. pylori *[38]; *M. tuberculosis *[39,40]; *S. aureus *[41,42]; *L. lactis *[43]), and of eukarya (e.g., Human [44]). The numerous mathematical tools have been used for i) identification and filling of knowledge gaps (e.g. missing gene annotations [45]); ii) prediction of the outcome of adaptive evolution [46-48]; iii) design of engineered production strains [49]; and iv) the understanding of topological features of metabolic networks [50-53]. A recent review illustrates the variety of questions that have been addressed to *E. coli*'s metabolic network using different biased and unbiased COBRA methods [54].

Here, we describe a highly detailed, genome-scale, metabolic reconstruction of *Pseudomonas putida *KT2440. Based on the naming convention for metabolic networks [55], this genome scale reconstruction was deemed *i*JN746, where *i* stands for *in silico*, JN are the initials of the constructor, and 746 corresponds to the number of included metabolic genes. The reconstruction was built using the COBRA approach [30,32] and validated using flux balance analysis (FBA, [56]). The *in silico *metabolic network was further evaluated by comparing i) predicted growth rate capacities in different carbon sources and ii) predicted essential genes with experimental data from *P. putida *KT2440 and *P. aeruginosa*. Finally, we show the utility of the *P. putida *reconstruction to analyze its biodegradative (i.e. toluene degradation) and biotechnological (i.e. bioplastic production) capacities.

## Results and discussion

### Characteristics of the metabolic reconstruction of *Pseudomonas putida *KT2440

The metabolic reconstruction of *P. putida *KT2440, *i*JN746, was constructed based on its annotated genome sequence [20], primary and review publications, various genetic and biochemical databases (i.e., KEGG Database [57], PSEUDOCYC [58], and SYSTOMONAS [59]), and biochemical information found in *Pseudomonas*-specific [21] and biochemical textbooks.

*i*JN746 accounts for 746 open reading frames (ORF), whose corresponding gene products are involved in 810 metabolic and transport reactions (Table 1). A total of 140 non-gene associated reactions were included in *i*JN746 based on physiological evidence in literature supporting their presence in *P. putida*'s metabolism. Hence, the reconstruction captures a total of 950 metabolic reactions and 911 metabolites distributed over three different cellular compartments: cytoplasm, periplasm, and extracellular space. Each metabolite was placed in one or more of these compartments depending on the cellular localization of the catalyzing enzyme, and the flux across outer and inner membranes was enabled by transport reactions.

**Table 1:** Properties of metabolic reconstruction of *P. putida *KT2440

***Reconstruction & Organism*** ****	***iJN746 *** ***P. putida***	***iAF1260 *** ***E. coli***	***iYO844 *** ***B. subtilis***	***iNJ661 *** ***M. tuberculosis***	***iMO1056 *** ***P. aeruginosa***
Protein coding genes per genome	5,350^a^	4,464^b^	4,106^a^	3,989^a^	5669
SKI value^c^	0.74	55.87	4.97	7.84	5.12
Genes (% of genome)	746 (14%)	1260 (28%)	844 (21%)	661 (17%)	1056 (18,6%)
Reactions	950	2077	1020	939	883
Gene-reaction associated	810	1919	904	723	839
Non-gene- associated network reaction (% of network reactions)	140 (17%)	158 (8%)	116 (13%)	116 (16%)	44 (5%)
Exchange reactions	90	304	225	88	-
Metabolites	911	1039	988	828	-

Properties of metabolic reconstruction of *P. putida *KT2440 were compared with recently published metabolic reconstructions of *E. coli *MG1655 (*i*AF1260 [36]), *B. subtilis *(*i*YO844 [37]), and *M. tuberculosis *H37Rv (*i*NJ661 [39]) and *P. aeruginosa* (iMO1056 [64]). ^a ^taken from KEGG [57]; ^b ^based on Riley et al. [98]; ^c ^Species Knowledge Index (SKI) was calculated as described in [65].

**Figure 1:**
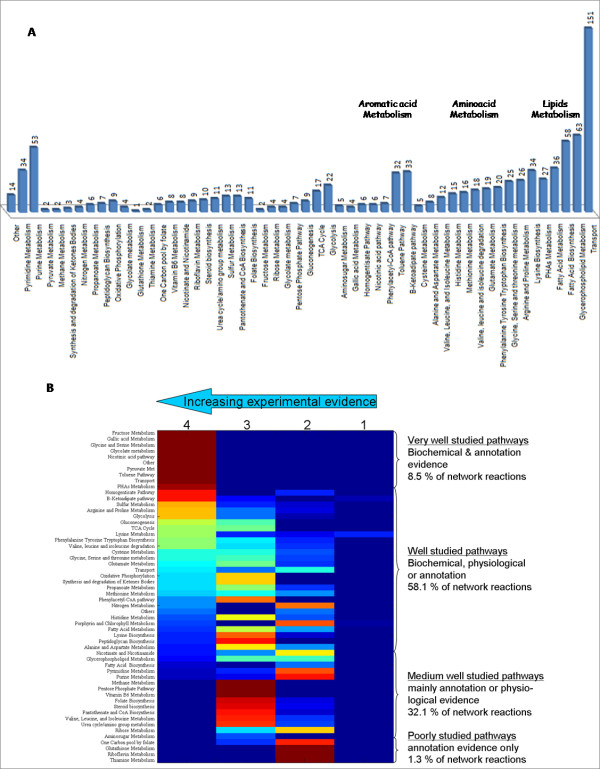
**A**. **Pie chart showing the distribution of *i*JN746's intracellular reactions over the different subsystems.** The number of reactions per subsystem is shown and subsystems of high importance were highlighted in bold. **B**. Heat map of the confidence score of the different subsystems in *i*JN746. The 4 rows in the map represent the different confidence score (from left to right: 4, 3, 2, 1). The various colors correspond to the percentage of subsystems reactions that have the corresponding confidence score (red = 100%, blue = 0%). The confidence level was based on a scale from 1 to 4. A level, or score, of 4 corresponds to biochemical evidence for a gene product and its reaction(s); 3 represents physiological, genetic, or proteomic evidence; 2 corresponds to only sequence-based evidence for a gene product and its reaction(s); and finally a score of 1 reflects that the reaction had to be included for model functionality (e. g., production of biomass precursor).

**Figure 2:**
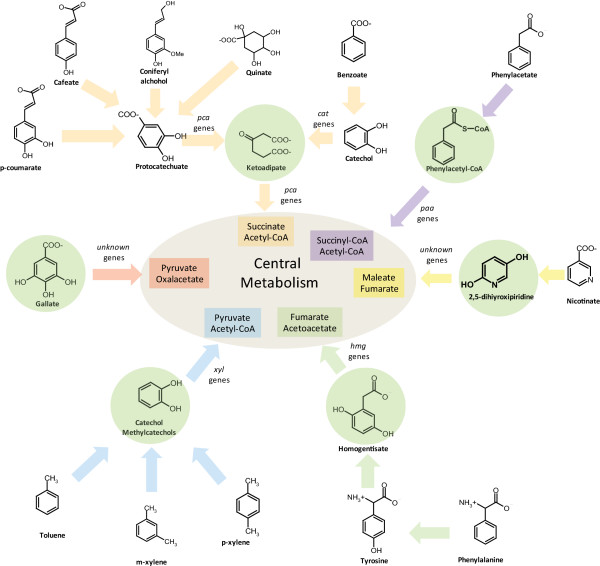
**General depiction of the aromatic compound degradation routes present in *i*JN746.** The protocatechuate (pca genes) and catechol (cat genes) branches of the beta-ketoadipate pathway are shown as well as peripheral pathways by orange arrows. The homogentisate pathway (hmg genes) is represented by green arrows and the phenylacetate pathway (paa genes) is represented by purple arrows. The nicotinate and gallate pathways (unknown genes) are shown by green and red arrows, respectively. Finally, the Tol pathway (xyl genes from pWW0 plasmid) for toluene and xylene degradation is represented by blue arrows. The initial aromatic compounds are indicated by green circles and the central metabolic compounds for each pathway are also highlighted. A detailed list of reactions involved in aromatic acid degradation can be found in the Additional file 9.

The reactions included in *i*JN746 were divided into 55 specific pathways, or subsystems, based on their functional role (Figure 1A). In general, the transport subsystem was found to be the subsystem with the highest number of gene-associated reactions, highlighting the importance of cellular transport for *P. putida*. This observation agrees well with the known lifestyle of *P. putida *[28] and successfully reflects the fact that approx. 12% of *P. putida *genome encodes for transport-associated gene products [20]. Reactions related to amino acid metabolism were also found to be very important since the de novo synthesis pathways for all 20 amino acids are present in *P. putida*'s genome [20]. Moreover, *P. putida *is known for its capability to utilize many amino acids as a carbon and nitrogen source [21,60]. A third group of great importance contained reactions involved in aromatic acid degradation pathways, which reflects the physiological ability of *P. putida *to use many of these compounds as a carbon and energy source (see Figure 2) [27]. Furthermore, despite the absence of the TOL pathway in KT2440's genome, the plasmid genes and the corresponding reactions were included into the *P. putida *metabolic reconstruction since the TOL plasmid is present in the parental strain *P. putida *mt-2 and this paradigmatic plasmid is often used to expand *P. putida *KT2440's metabolic capacities [6,12]. Finally, reactions associated with lipid metabolism constituted another important subsystem group. In fact, *P. putida *KT2440 can synthesize and accumulate medium-side-chain polyhydroxyalkanoates (msc-PHAs), which are lipid related polymers, from a wide range of carbon sources [17,61]. This ability is of special interest for biotechnological purposes (reviewed in [62,63]) and therefore, we incorporated both the msc-PHAs biosynthetic and TOL biodegradative pathways into the metabolic reconstruction (see below).

Every network reaction was associated with confidence scores based on the available evidence for its presence in the *P. putida *metabolic network (Figure 1B). For instance, reactions whose enzymes have been biochemically studied in *P. putida *received a confidence score of 4. If physiological or genetic knockout information was available, a score of 3 was associated with the network reaction. Reactions associated with enzymes that were only annotated in *P. putida*'s genome but had no further experimental evidence were given a confidence score of 2. Finally, during the evaluation of the network functionality (i.e. biomass precursor production) some reactions had to be added to the network for which no genetic or experimental evidence could be found. Those reactions represent modeling hypotheses, which need further experimental validation and thus received a confidence score of 1. Upon completion, the reconstruction had an overall average confidence score of 2.83. In fact, two thirds of *P. putida*'s metabolic pathways have been very well or well studied, while only a third of the subsystems were primarily based on the genome annotation (Figure 1B). This high level of confidence is also reflected by the number of references that lead to this metabolic reconstruction. Almost 90% of the internal reactions (844) have at least one associated citation, while a total of 176 unique primary and review publications were reviewed and incorporated into this reconstruction. Subsequently, this first genome-scale reconstruction of *P. putida*'s metabolism represents a comprehensive knowledge base summarizing and categorizing the information currently available. The content of this knowledge base will be easily accessible through the BiGG database .

### Comparison of scope and content of *i*JN746 with published metabolic networks

The properties of *i*JN746 were compared with the properties of recently published reconstructions of *E. coli *MG1655 (*i*AF1260, [36]), *B. subtilis *(*i*YO844 [37]), *M. tuberculosis *H37Rv (*i*NJ661 [39]), and *P. aeruginosa *PAO1 (*i*MO1056 [64] (Table 1). We found that the percentage of included ORFs was smaller in *i*JN746 than in the other reconstructions. Subsequently, it can be expected that the number of metabolic functions present in *P. putida *is larger than currently identified in the genome annotation and literature. In fact, the number of included non-gene associated reactions was twice that of the *E. coli *metabolic reconstruction. Furthermore, the species knowledge index (SKI) [65], which relates the number of PubMed abstracts of an organism to its number of ORFs, was much lower for *P. putida *compared to the other reconstructions. In summary, this comparison indicates that the overall context coverage in *i*JN746 is comparable with other high-quality network reconstructions when the amount of available literature is considered.

A metabolic reconstruction for another representative of the *Pseudomonas *genera was published recently [64]. A comparison of *P. putida *and *P. aeruginosa *metabolic reconstructions was performed (Table 1). In contrast to *P. putida*, *P. aeruginosa *is an opportunistic human pathogen and as such more information about its metabolism and physiology is available, which is directly reflected by a SKI value 7 times higher than that of *P. putida *(Table 1). As a consequence, a larger number of metabolic genes were included in the metabolic reconstruction (14% of *P. putida*'s genome vs. 18% of *P. aeruginosa*'s genome). Despite being close relatives, these two representatives have significant differences in lifestyle and metabolic capabilities. Subsequently, the two metabolic reconstructions have significant differences, emphasizing the importance of organism-specific reconstructions. For instance, the *P. aeruginosa *reconstruction contains pathways necessary for growth and production of common virulence factors, including alginate, rhamnolipids, phenezines, and quorum-sensing molecules [64], which are not present in *P. putida*'s metabolic network. In contrast, *P. aeruginosa*'s metabolic network does not account for pathways necessary to degrade aromatic compounds.

### *i*JN746's metabolic versatility

Flux balance analysis (FBA [56]) can provide insight into the growth capabilities of the reconstructed network. Comparison of *in silico *growth performance with experimental data allows for the assessment of the predictive potential of the metabolic reconstruction and thus represents a valuable tool for network evaluation. Furthermore, *in silico *growth analysis may expand the known array of carbon-, nitrogen-, and energy sources of the reconstructed organism. In this study, the aerobic growth capabilities of *i*JN746 in *i*M9 medium substituted with different carbon sources were determined qualitatively (Table 2) and quantitatively (Table 3). The growth simulation results reflected the metabolic versatility for which *P. putida *is well known, with a total of 59 carbon sources enabling *in silico *growth when added to the *i*M9 minimal medium (Table 2). Furthermore, we compared the *in silico *growth performance on different carbon, sulfur, and nitrogen sources with phenotyping data derived from literature [see Additional file 1]. For instance, *P. putida *is found in terrestrial and aquatic environments around the world, with preference for the rhizosphere [21], which is especially rich in carbon sources, amino acids, organic acids, and aromatic acids derived from seeds, roots, and other plant parts [66,67]. This niche specificity accounts for the broad carbon source usage of KT2440 and therefore, most of the known soil carbon sources were captured in *i*JN746 (Table 2). Of particular biotechnological importance is the ability of *i*JN746 to metabolize aromatic compounds, thus, representing the first metabolic reconstruction accounting for growth on these carbon sources. For example, aromatic compounds such as toluene or xylene are of special interest as they are archetypical pollutants. Subsequently, we studied the toluene degradation process using *i*JN746 (see below).

**Table 2:** Carbon sources enabling growth of *i*JN746 in *i*M9 mineral medium.

***Class***	***Compound***	***Class***	***Compound***
Aromatic and related compounds		Amino acids	
			
	Protocatechuate		L-Arginine
	Caffeate		L-Aspartate
	Oxoadipate		L-Glutamate
	4-Hydroxybenzoate		L-Glycine
	Benzoate		L-Histidine
	Catechol		L-Isoleucine
	Coniferyl alcohol		L-Leucine
	Ferulate		L-Lysine
	Gallate		L-Proline
	m-Xylene		L-Serine
	Nicotinate		L-Threonine
	p-Xylene		L-Valine
	Phenylacetate	Organics acids	
			
	L-Phenylalanine		α-Ketoglutarate
	Quinate		Citrate
	p-Coumarate		Fumarate
	Toluene		Isocitrate
	L-Tyrosine		D-Lactate
	Vanillin		L-Lactate
	Vanillate		Malate
Fatty acids			Succinate
			
	Acetate	Carbohydrates	
			
	Decanoate		2-ketogluconate
	Dodecanoate		D-Fructose
	Hexadecanoate		D-Glucose
	Hexanoate		D-Gluconate
	Octanoate		D-Ribose
	Propionate	Miscellaneous compounds	
			
	Tetradecanoate		4-Aminobuturate
Polyalcohols and glycols			Glycine betaine
			
	Glyceraldehyde		Ornithine
	Glycerol		Choline
	Glycolate		Choline sulfate

Carbon sources enabling growth of *i*JN746 in *i*M9 mineral medium. The compounds were grouped based their structural characteristics. A complete list of carbon sources tested, along with possible nitrogen and sulfur sources, as well as bibliographic support can be found in the Additional file 2.

No false positive carbon, nitrogen, or sulfur sources were found in *i*JN746, as expected, as only exchange reactions were included in the reconstruction for metabolites, which have been reported to be taken up or secreted by *P. putida *KT2440. In contrast, some disagreements, such as false negatives, were observed despite a good overall agreement with the *in vivo *data [68] [Additional file 1]. For example, it was reported that *P. putida *can use L-alanine as a carbon- and nitrogen-source [68] but *i*JN746 cannot use this compound as a carbon or nitrogen source. This disagreement could not be resolved. In contrast, *i*JN746 was initially unable to use choline-O-sulphate, choline, or glycine betaine as carbon- and nitrogen-sources despite experimental evidence [69]. However, the addition of two non-gene-associated reactions, betaine-homocysteine S-methyltransferase (EC- 2.1.1.5) and dimethylglycine dehydrogenase (EC- 1.5.99.2), enabled *i*JN746 to use these metabolites as carbon- and nitrogen-sources through the glycine metabolism. In addition, choline-O-sulphate could also be used as sulfur source [see Additional file 1]. The two added reactions represent a hypothesis that needs further experimental verification. These examples show how discrepancies between *in silico *predictions and physiological properties can be used to drive new discoveries, as was shown for *E. coli *[45].

### Growth on glucose

**Figure 3:**
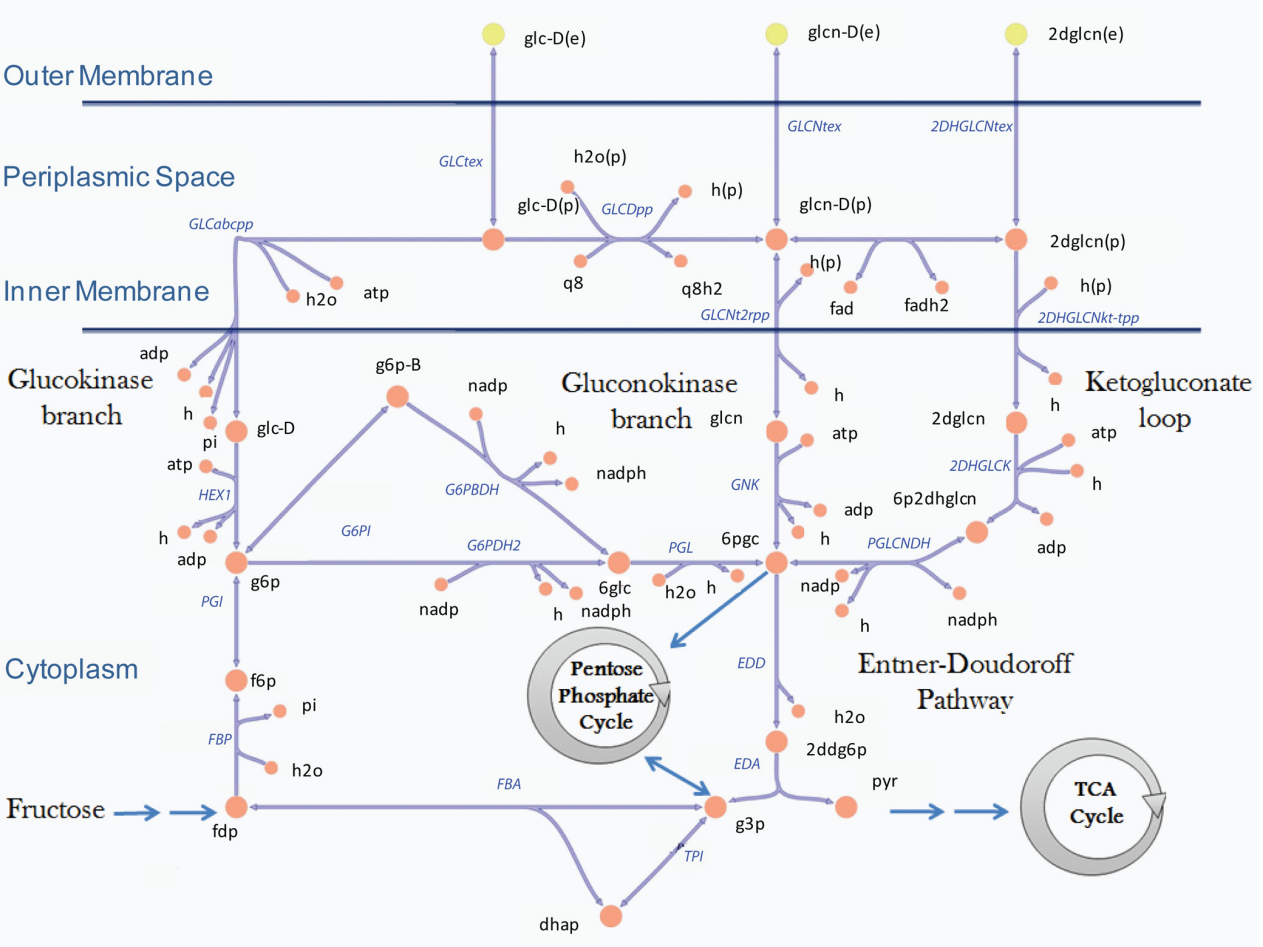
Glucose metabolizing pathways present in *P. putida *KT2440 and its metabolic reconstruction, *i*JN746.

*P. putida *KT2440, like other *Pseudomonas *species and rhizosymbionts, has an incomplete glycolytic pathway because of a missing 6-phosphofructokinase [70]. However, *P. putida *KT2440 has a complete Entner-Doudoroff pathway, which allows for the utilization of glucose and other sugars as carbon sources (Table 2). Therefore, we investigated the properties of glucose metabolism in *i*JN746 to validate and evaluate the reconstructed network [71]. For instance, comparison of predicted *in silico *growth with experimental data permits a direct assessment of the predictive potential of a reconstructed metabolic network. Subsequently, we determined the aerobic growth capability of *i*JN746 in Glucose-M9 minimal medium (*i*M9). Interestingly, *i*JN746 grew faster in glucose than experimental *in vivo *data suggested for *P. putida *KT2442 (Table 3, [25]). A similar difference in growth rate between *in vivo *and *in silico *measurements was reported for *P. aeruginosa *[64]. The difference in growth rate might be explained by an incomplete formulation of biomass function or higher energy maintenance requirements not accounted for in the current reconstruction [30,36] or missing adaptation to glucose as primary carbon source. Another explanation could be that *P. putida *KT2442 converts only a part of glucose into biomass. In fact, a recent study showed that *P. putida *KT2442 accumulated low, extracellular concentrations of gluconate and 2-ketogluconate when grown on glucose [25]. *P. putida *metabolizes glucose exclusively via the Entner-Doudoroff pathway in which 6-phosphogluconate is the key intermediate. This compound is produced by three convergent pathways; the glucokinase branch, the gluconokinase branch, and the 2-ketogluconate loop (Figure 3)[70]. The latter two pathways produce gluconate and 2-ketogluconate as intermediate compounds of the glucose catabolism. *i*JN746 accounts for these alternate routes and corresponding transport reactions for gluconate and 2-ketogluconate.

**Table 3:** Comparison of growth performance of the *in silico *strain *i*JN746 and KT2442.

**Strain**	**Carbon ** **source**	**μmax(h^-1^) ** **iJN746**	**μmax(h^-1^) ** **KT2442**	**Carbon Uptake rate ** **(mmol gDW/h)**	**O_2 _Uptake rate ** **(mmol gDW/h)**
*i*JN746/KT2442	Glucose	0.751	0.56a	6.3a	15.34d
*i*JN746/KT2442	Toluene	0.421	0.72b	11.9b	18.5c
*i*JN746/KT2442	Toluene	0.476	0.72	11.9	20.93d
*i*JN746/KT2442	Toluene	0.7255	0.72	11.9	33
*i*JN746/KT2442	Toluene	1.262	0.72	11.9	∞

Comparison of growth performance of the *in silico *strain *i*JN746 and KT2442. The *in silico *growth rate was calculated in *i*M9 minimal medium plus glucose or toluene. Due to candidate oxygen limited growth in toluene, the *in silico *growth rate was calculated under different oxygen uptake rates In addition *i*JN746 growth in toluene as only carbon source was simulated at different oxygen uptake rates. ^a ^from [25]; ^b ^from[26]; ^c ^from [78], and ^d ^experimentally determined in this study.

### Growth on Toluene

Aromatic compounds such as toluene or xylene are found in polluted soil. Some *Pseudomonas *species are known to grow on these compounds as a sole carbon source [72], making them interesting candidates for bioremediation of contaminated areas [9,10]. As indicated above, *P. putida *KT2440 can metabolize various aromatic acids, amino acids, sugars, organic acids, fatty acids, and organo-sulfur compounds (see Table 2). More specifically, *P. putida *KT2440 degrades many aromatic compounds into a limited number of intermediates using a few catabolic pathways that were captured in *i*JN746 (Figure 2). In particular, the toluene biodegradation pathway has been extensively studied in *P. putida *[73-75] and its genetic regulation is well known [76]. In this study, we assessed the capability of *i*JN746 to quantitatively predict aerobic growth on toluene (Table 3). The comparison showed a much lower *in silico *growth rate when compared to *in vivo *data, 0.421 versus 0.72 (60%) (Table 3). In the following, we used different mathematical tools to elucidate reasons for this significant discrepancy.

#### Reduced cost of toluene catabolism

Linear Programming (LP) problems have two parameters, shadow price and reduced cost, which can be used to characterize the optimal solution. While shadow prices are associated with each network metabolite, reduced costs are associated with each network reaction. The reduced cost signifies the amount by which the objective function (e.g. growth rate) would increase when the flux rate through a chosen reaction was increased by a single unit [77]. Analyses of the reduced costs associated with uptake rates in the oxygen-limited toluene simulations identified the OUR as the only non-zero reduced cost value, 0.021 g biomass/gDW/h. This value corresponds to an increase of the OUR to 33 mmol oxygen/gDW/h to achieve the experimentally determined growth rate [26]. At an OUR higher than 62 mmol oxygen/gDW/h oxygen is no longer a growth-limiting factor but toluene is. Note that the upper limit of 18.5 mmol oxygen/gDW/h for the OUR was taken from measurements for *E. coli *corresponding to the normal oxygen diffusion rate under atmospheric oxygen conditions [78]. Mathematically, the reduced cost analysis supports the hypothesis that oxygen is the limiting factor for toluene catabolism and hence causes the reduced *in silico *growth rate.

#### Phase Plane Analysis of toluene catabolism and oxygen uptake

**Figure 4:**
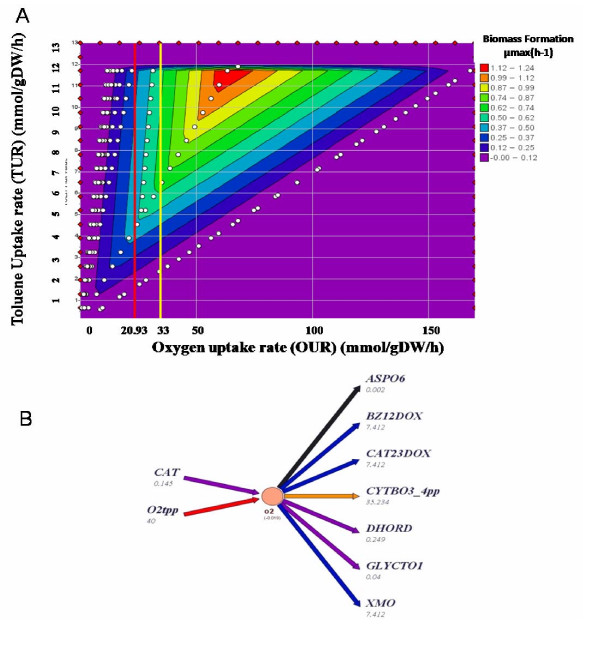
**(A) The phenotypic phase plane analysis showed growth rate as a function of OUR and TUR in *****i*****JN746.** The growth rate is given in 1/h (color legend). The red and yellow lines represent OUR constrained to 20.93 and 33 mmol/gDW/h, respectively. **(B) **Diagram of oxygen producing and reducing reactions in *i*JN746. The flux rates are given in mmol/gDW/h and represent one possible flux state of the network in toluene minimal medium at an OUR of 40 mmol oxygen/gDW/h. The reaction abbreviations are as follows: CAT, catalase; O2tpp, oxygen periplasmic transport (oxygen uptake); ASPO6, L-aspartate oxidase; BZ12DOX, benzoate 1,2-dioxygenase; CAT23DOX, catechol 2,3-dioxygenase; CYTBO3_4pp, cytochrome oxidase bo3; DHORD, dihydoorotic acid dehydrogenase; GLYCTO1, Glycolate oxidase and XMO, toluene monooxygenase.

We performed a phase plane analysis to further elucidate the correlation between toluene uptake, OUR, and biomass production rate (Figure 4). We analyzed all four cases listed in Table 3 and found a direct effect of increased OUR on the toluene uptake capability and biomass production rate (Figure 4A). The experimentally observed growth rate of 0.72 μmax(h^-1^) [26] was achieved by TUR ranging from 6 to 11.9 mmol toluene/gDW/h and OUR higher than 33 mmol oxygen/gDW/h. Note that a higher toluene uptake rate (TUR) requires a higher OUR (Figure 4A), which indicates that the removal of intracellular oxygen was dependent on toluene availability. In fact, the three oxidative reactions involved in the conversion of toluene to 2-hydroxymuconate semialdehyde (toluene monooxygenase, benzoate 1,2-dioxygenase and catechol 2,3-dioxygenase) were found to have the higher flux rates besides the flux through the cytochrome C oxidase, an enzyme of the oxidative phosphorylation (Figure 4B).

In order to better understand this situation and since no detailed information about OUR was found for *P. putida *KT2440 under toluene-dependent growth conditions, we carried out *in vivo *experiments to determine the OUR of *P. putida *KT2440 harboring the TOL plasmid (see Methods). As expected, the OUR in toluene growing cells was higher than glucose or octanoate growing cells; 20.93 compared to 15.34 and 14.88 mmol oxygen/gDW/h, respectively (Table 3). The measured OUR uptake rate for growth in toluene did not explain the high oxygen requirement of the model, but clearly indicates the importance of oxygen uptake in toluene metabolism. Also, the measured OUR was slightly higher than the *E. coli *value that was used for the standard *in silico *simulations (20.93 vs. 18.5 mmol oxygen/gDW/h). In fact, oxygen dependent growth of toluene grown cells has been described for other *P. putida *strains. For example, Alagappan and Cowan reported a 10× higher oxygen-half saturation of *P. putida *F1 grown on toluene than other aerobic organisms [79]. Furthermore, the oxidative stress caused by toluene and other aromatic acids in the degradative process is well known [23,80]; however, this phenomenon was found to be mainly caused by reactive oxygen species due to incomplete oxygen reduction [81], indicating an active oxygen metabolism under this growth condition. Oxygen-limiting growth conditions were also reported for *P. putida *when grown on octanoate [63].

Taken together, our analysis suggests that the current *P. putida *metabolic network is incomplete. In fact, the current information and results suggest that the network is missing one or more reactions enabling a more oxygen-efficient catabolism of toluene and other highly reduced carbon sources (e.g. other aromatic compounds or fatty acids). This analysis represents a nice example of the broad range of applications for which *i*JN746 can be used to evaluate the consistency of experimental data and *in silico *prediction. *i*JN746 can serve as a platform to derive hypotheses about metabolic capabilities or missing functions in the network which can be ultimately tested in the laboratory. Hence, the metabolic reconstruction can help to increase our understanding and knowledge about this biotechnologically important organism.

### Gene essentiality analysis in *i*JN746

*i*JN746 was used as a framework to analyze candidate essential genes in *P. putida *KT2440 in LB rich medium. Therefore, the network reaction(s) associated with each gene was individually "deleted" by setting the flux to 0 and optimizing for the biomass function [32]. We wished to compare the *in silico *essentiality predictions with experimental data to assess the predictive potential of the model. However, no large-scale, experimental gene essentiality data are available for *P. putida*; the information can only be found for its phylogenetic relative *P. aeruginosa *PAO1 and *P. aeruginosa *PA14 [82,83]. A recently published comparison between the *P. putida *and *P. aeruginosa *PAO1 genomes identified 3,143 potential orthologous pairs corresponding to 60% of *P. putida*'s total ORFs, as well as large sections of conserved gene order (synteny) [28]. Therefore, we decided to compare our *in silico *single gene deletion results with the 335 essential metabolic and non-metabolic genes of *P. aeruginosa *[82,83]. About 12% (92) of the 746 metabolic genes present in *i*JN746 were predicted to be essential in *i*LB medium [see Additional file 2]. A total of 53% (48) of these predicted essential genes in *i*JN746 agreed with essential genes of *P. aeruginosa *[see Additional file 3]. More importantly, the 44 genes wrongly predicted as essential genes represent excellent targets for further refinement and expansion of the metabolism of *i*JN746 [see Additional file 4] as has been done for *E. coli *[45].

#### False-positive predictions

The disagreement between the experimental and computational results can reveal possible errors in the experimental data as well as in the reconstructed network. The disagreements might be caused by low experimental or sequence evidences, each of which would have hindered the inclusion of the information into the reconstruction. For example, the *fabB *gene was predicted to be only essential in *i*JN746; however, after carrying out a detailed search on *Pseudomona's *genomes using "The *Pseudomonas *Genome Database V2"  we found putative ORFs in the KT2440 and PA01 genome. These ORFs were annotated as alternative loci that could substitute a *fabB *deletion. Both, *P. putida *and *P. aeruginosa *have one copy of the *fabB *gene encoding for the 3-oxoacyl-(acyl-carrier-protein)synthase I (PP_4175 and PA1609, respectively). In addition, both strains have a copy of the *fabF *gene encoding for the 3-oxoacyl-(acyl-carrier-protein) synthase II (PP_1916 (40.92% identity with *fabB*-_KT _gene) and PA2965 (42.34% identity with *fabB*-_PAO1 _gene). Moreover, in the *P. putida *and *P. aeruginosa *genome, some ORFs were annotated putatively to encode for a 3-oxoacyl-(acyl-carrier-protein) synthase II (PP_3303 (35.94% identity) and PP_2780 (27.32% identity) in KT2440, and PA_1373 (36.17% identity) in PAO1 strain. These putative ORFs were not included in *i*JN746 due to the lack of supporting evidence for their metabolic function, but this analysis showed that i) PAO1 has an isozyme present in its genome, and ii) KT2440 is very likely to have at least one other ORF encoding this or a similar function. In a similar way, the discrepancy between *in silico *essentiality prediction and *in vitro *observation for *msbA *gene could be explained. The gene product of *msbA *encodes for a transporter of phosphatidylethanolamine, which is known to have a genetic redundancy in *Pseudomonas sp*. taking into account the *Pseudomonas *annotation present in "The *Pseudomonas *Genome Database V2". However, the supporting evidence for alternative ORFs was not strong enough to be included into *i*JN746.

Finally, 37 genes were not predicted to be essential in *i*JN746 but they were reported as essential genes in *P. aeruginosa *[83] [see Additional files 4 and 3]. Of these false negatives, 13 genes encode for tRNAs synthetases which are typically included into metabolic networks [36] but are not functionally connected to the rest of the network. Hence, this disagreement was expected. Four additional false negative predictions, namely *glyA *(PP_0322 or PP_0671), *fold *(PP_1945 or PP_2265), *fabZ *(PP_4174 or PP_1602), and *pyrH *(PP_1771 or 1593), have at least one isozyme in KT2440 which were also accounted for in *i*JN746. For many remaining incorrectly predicted non-essential genes, the *in silico *deletion had a significant effect on the growth rate, reflecting their important roles in *i*JN746 metabolism [see Additional file 5].

In general, many of these discrepancies suggest that metabolites enabling growth in the knock-outs might be imported from the external rich media since the exact composition of LB medium is not known [37,38]. This observation indicates the importance of using well defined minimal media in the experimental *in vivo *or *in vitro *procedure to enable the usage of the generated data for *in silico *predictions and comparison.

#### Gene essentiality and amino acid auxotrophy

Jacobs *et al*. reported a detailed amino acid auxotroph study in *P. aeruginosa *PA01 using a minimal medium [82]. We carried out another single gene deletion study in glucose *i*M9 medium and compared the results with this PA01 study. Here, we found an absolute agreement between *in vivo *and *in silico *gene essentiality for six amino acids, namely arginine, histidine, isoleucine, valine, leucine, and tryptophan (Table 4). The presence of alternative loci in *i*JN746 explains partial disagreement for *argA*, *argE*, *ilvA*, and *argJ*. In fact, genetic redundancy for these genes was reported in *Pseudomonas *species [82]. This high correlation between *in silico *and *in vivo *data shows the utility of this approach when you take into account metabolic or anabolic reactions in a well defined minimal media. The complete lists of potential essential genes predicted in glucose *i*M9 medium are listed in the Additional file 6.

**Table 4:** The comparison of the *in silico *gene essentiality and experimental *P. aeruginosa *data are shown under various amino acid auxotrophic conditions.

**Amino acid**	**PP gene**	**gene**	**Reaction**	**iJN746/PA01€ (growth)**
Arginine	PP_5185(PP_1346)	argA†,(argJ)	ACGS,(ORNTAC, ACGS)	(+/-)*
				
	PP_5289	argB	ACGK	(-/-)
	PP_3633	argC	AGPR	(-/-)
	PP_5186,(PP_1346)	argE†,(argJ)	ACODA(ORNTAC, ACGS)	(+/-)*
	PP_1088	argG	ARGSS	(-/-)
	PP_0184	argH	ARGSL	(-/-)
	PP_1346	argJ†	ORNTAC, ACGS	(+/-)*
Histidine				
				
	PP_0292	hisA	PRMICIi	(-/-)
	PP_0289	hisB	IGPDH	(-/-)
	PP_0967	hisC	HSTPTr	(-/-)
	PP_0966	hisD	HISTD	(-/-)
	PP_5015	hisE	PRATPP	(-/-)
	PP_0293	hisF	IG3PS	(-/-)
	PP_0965	hisG	ATPPRTr	(-/-)
	PP_0290	hisH	IG3PS	(-/-)
	PP_5014	hisI	PRAMPC	(-/-)
Isoleucine-valine				
				
	PP_3446, PP5149	ilvA-1, ilvA-2	SER_AL, THRD_L	(+/-)*
	PP_4680	ilvB (ilvI)£	ACHBS, ACLS	(-/-)
	PP_4678	ilvC	KARA1, KARA2	(-/-)
	PP_5128	ilvD	DHAD1, DHAD2	(-/-)
	PP_3511	ilvE	VALTA, LEUTA, ILETA	(-/-)
	PP_4679	ilvN(ilvH)£	ACHBS, ACLS	(-/-)
Leucine				
				
	PP_1025	leuA	IPPS	(-/-)
	PP_1988	leuB	IPMDr	(-/-)
	PP_1985	leuC	IPPMIa, IPPMIb	(-/-)
	PP_1986	leuD	IPPMIa, IPPMIb	(-/-)
Tryptophan				
				
	PP_0082	trpA	TRPS1r, TRPS3r	(-/-)
	PP_0083	trpB	TRPS2, TRPS1r	(-/-)
	PP_0422	trpC	IGPS	(-/-)
	PP_0421	trpD	ANPRT	(-/-)
	PP_0417	trpE	ANS	(-/-)
	PP_1995	trpF	PRAI	(-/-)
	PP_0420	trpG	ANS	(-/-)

The comparison of the *in silico *gene essentiality and experimental *P. aeruginosa *data are shown under various amino acid auxotrophic conditions. The *in silico *mutants were grown on Glucose-iM9 medium. * No auxotrophy was detected in *i*JN746, genetic redundancy for these genes was reported in *Pseudomonas *species. In *P. aeruginosa *mutants for orthologous genes, a significant residual growth on minimal medium was shown [82]. £ Alternative name in *P. aeruginosa*. € From [82].

### *i*JN746 as a cell factory

In the previous section, we used the metabolic reconstruction to assess the current knowledge of *P. putida's *metabolism by comparing and testing *in silico *predictions with physiological data. However, metabolic network reconstructions can also serve as engineering and design tools [49] in addition to their use for discovery purposes [45]. Here, we investigate the poly-3-hydroxyalkanoate (PHA) production capability by the metabolic network. PHAs are a class of microbially produced polyesters that have the potential to replace conventional, petrochemically derived plastics in packaging and coating applications [63]. The biotechnological interest originates from their biodegradability and the broad range of physical properties depending on the number of carbons and side chains present in the PHA polymers [63]. These polymers are stored by many microorganisms under inorganic nutrient limited and carbon-excess growth conditions and are used as carbon- and energy sources under starvation conditions [63]. The medium-side-chain PHAs (msc-PHAs) are composed of C_6 _to C_16 _3-hydroxy fatty acids and are commonly produced by fluorescent *Pseudomonas*. In this way, *P. putida *KT2440 is an excellent candidate for msc-PHA production studies, since i) the basic msc-PHA production processes in KT2440 are well known [17,61], ii) its genome is completely sequenced, iii) KT2440 has a well known metabolic versatility (can use a large list of carbon source as PHA precursors), iv) it is a very good host-vector biosafety system for gene cloning and expression of heterologous genes and v) this strain has been used in numerous biotechnology processes including msc-PHA production.

**Figure 5:**
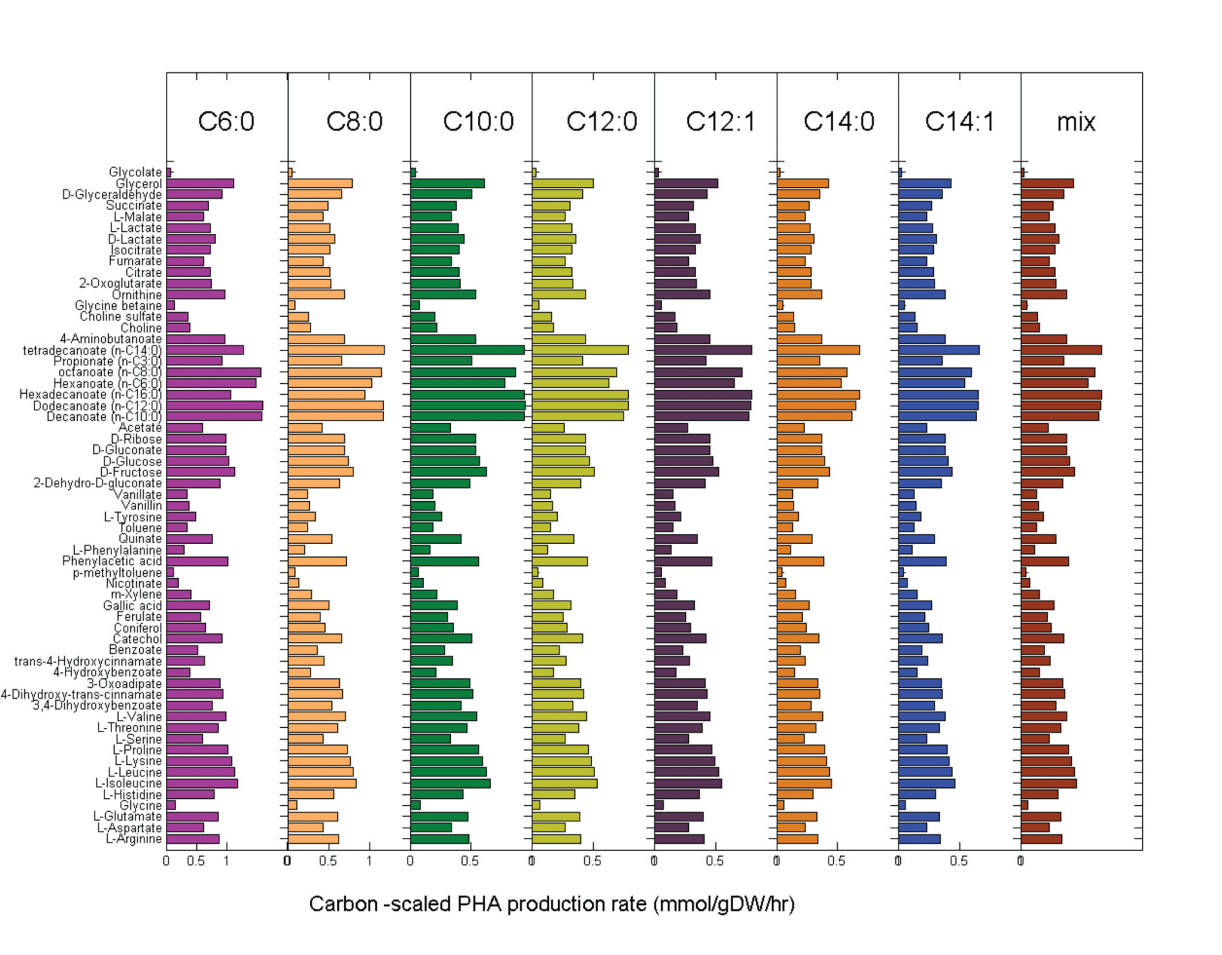
**Maximal possible msc-PHA production rate from various carbon sources**. The msc-PHA production rate is scaled per substrate carbon to facilitate the yield comparison. The simulation conditions correspond to chemostate culturing at a dilution rate of μ = 0.2 1/hr, minimal medium (iM9) supplemented with each carbon source. 'Mix' corresponds to the simultaneous production of C8:0, C6:0, C10:0, and C12:0 msc-PHA (1:1:1:1).

*i*JN746 accounts for msc-PHAs ranging from C_6 _to C_14_, including two unsaturated msc-PHAs and a mixed msc-PHA polymer consisting of C_8 _to C_12 _chains. We tested the msc-PHA production capability of *i*JN746 from the different carbon- and energy sources listed in Table 2. All carbon sources were found to result in msc-PHA production under the chosen simulation condition (dilution rate of 0.2 hr^-1^). Many of these metabolites have been reported to yield in PHA production in *Pseudomonas *[see Additional file 7] although many studies focused on fatty acid or carbohydrate derived msc-PHAs. In general, it is assumed that carbon sources generating high levels of acetyl-CoA are good candidates for PHA production [63]. Therefore, it was not surprising to find fatty acids and carbohydrates as the best PHA precursors in *i*JN746 as well (Figure 5). The list of candidate (*in silico*) precursors includes i) L-branched-chain amino acids (L-leucine, L-isoleucine, L-Valine etc), ii) some aromatic compounds metabolized via β-ketoadipate pathway (catechol, p-coumarate, etc), and iii) other (phenylacetic acid or glycerol) (Figure 5). Interestingly, phenylacetic acid and glycerol have been reported as excellent precursors for PHA [Additional file 7]. In fact, a recent study showed that *P. putida *CA3 can accumulate 0.17 g of PHA per g of phenylacetate [84].

Fatty acids resulted in the highest PHA production rate overall and when scaled per carbons (see Figure 5, and Additional file 7). In fact, fatty acids are converted into msc-PHAs quickly via β-oxidation [63]. Experimental studies showed that the resulting msc-PHA-monomers have the same or a smaller number of carbons as the fatty acids from which they are derived [61,85]. In contrast, in the model, higher carbon msc-PHAs could be formed since the current model formulation does not exclude simultaneous fatty acid synthesis and β-oxidation. This situation has been experimentally demonstrated using hexanoate as a msc-PHA precursor. Huijberts *et al*. used inhibitors of fatty acid metabolism and demonstrated that, depending on the nature of the substrate, precursors for PHA synthesis could be derived from either beta-oxidation or fatty acid biosynthesis, and interestingly, when hexanoate was used as carbon source for msc-PHA accumulation, both routes can operate simultaneously [61]. On the other hand, the carbohydrates are converted into msc-PHA from intermediates of the fatty acid synthesis and have been shown to result primarily in C_8 _and C_10 _monomers. The model, in contrast, is able to produce the full range of msc-PHAs from carbohydrates (Figure 5). These discrepancies suggest that despite broad specificity of the Poly-(3-hydroxyalkanoate) polymerase, ranging from C_6 _to C_16 _3-hydroxy fatty acids [17], the PHA polymerizing enzyme system might have preferences for monomers with 8 or 10 carbon atoms, while larger and smaller monomers are incorporated less efficiently. This fact can also explain why, during growth on hexanoate, msc-PHA precursors are synthesized by elongation and *de novo *fatty acid synthesis pathway, resulting more preferably in the generation of C8 and C10 monomers [61]. Such differences in specific activity could be applied as additional constraints to the model to obtain similar results as those observed experimentally.

Taken together, this example illustrates how *i*JN746 could be employed as a tool to identify new substrates (catechol, p-coumarate, isoleucine etc) for production of the different msc-PHA monomers or msc-PHA mixtures. Furthermore, computational tools such as OptKnock[86] or OptStrain[87] could help to design i) higher production strains, and/or ii) couple PHA production to growth rate. Such approaches have proven successful for other metabolic engineering designs such as lactate production in *E. coli *[88] or succinate production in *M. succiniciproducens *[89].

## Conclusion

Here, we presented the first genome-scale reconstruction of *P. putida*, a biotechnologically interesting all-surrounder.* i*JN746 is a highly detailed reconstruction of the *P. putida *KT2440 metabolic network that captures the important biotechnological capabilities, such as biodegradation of aromatic compounds, of this paradigmatic bacterium. Moreover, *i*JN746 represents a comprehensive knowledge base summarizing and categorizing the information currently available for *P. putida *KT2440. This study evaluated the metabolic network content and showed some examples of how *i*JN746 could be used for biotechnological purposes. Taken together, our results underlined the value of *i*JN746 as a suitable tool to study of *P. putida*'s metabolism and its biotechnical applications by the *P. putida *community.

## Methods

### *In vivo *determination of oxygen consumption and cell culture condition

*P. putida *KT2440 harboring the TOL plasmid was used for *in vivo *determination of oxygen consumption experiments. The bacterium was grown at 30°C in M9 minimal medium [90] with octanoate (15 mM), glucose (0.3% [wt/vol]), or toluene (6 mM) as a carbon source. Liquid cultures were agitated on a gyratory shaker operated at 250 rpm. For the OUR experiment, an overnight culture of *P. putida *KT2440 strain grown in each carbon source was diluted until the turbidity at 600 nm (OD_600_) was 0.05 in fresh M9 minimal medium with the appropriate carbon source, samples were then incubated until the culture reached a turbidity at 600 nm of 0.6 for glucose or octanoate growing cells and 0.45 in toluene growing cells. Aliquots of 2 ml were taken for OUR determination; the cells were harvested by centrifugation, washed twice and re-suspended in 1 ml of fresh medium containing the appropriate carbon source using the above concentrations. The OUR was measured by monitoring the substrate-dependent oxygen consumption rate at 30°C using an oxygen electrode (DW1 Hansa-Tech Oxygen Electrode, Hansa-Tech Oxygen Instrument Limited) in 1-ml assay mixture. Cellular dry weight (CDW) was determined using previously published methods [91], using at least 3 parallel 10-ml cell suspensions that were harvested by centrifugation at 15,800 × *g*. The pellets were washed with 0.9% NaCl and then dried at 105°C for 24 h to a constant weight using pre-dried and weighed 2-ml Eppendorf cups.

### Network reconstruction

The reconstruction process was done as described previously [30]. Briefly, the genome annotation of *P. putida *KT2440 was obtained from TIGR (, 06/27/2007) and was used as the framework of the network reconstruction. *P. putida*-specific primary and review literature and books were used to retrieve information about every network reaction: i) substrate specificity, ii) coenzyme specificity, iii) reaction directionality, iv) enzyme and reaction localization, and v) gene-protein-reaction (GPR) association. Relevant references were associated with every network reaction [see Additional files 7 and 8]. Public databases such as KEGG [57], PSEUDOCYC [58], and SYSTOMONAS [59] were used when no literature evidence could be found for the previous reaction characteristics. Spontaneous reactions were included into the reconstruction if i) physiological evidence suggested their presence (e.g., the presence of at least the substrate or product in the reconstruction); and ii) textbooks or KEGG [57] suggested the existence of such reactions. Every network reaction was mass- and charge balanced assuming an intracellular pH of 7.2 [38,55]. Note that this mass- and charge balancing also included balancing the network reactions for protons (H^+^), water (H_2_O), and various co-factors (e.g., adenosine triphosphate (ATP)). No gene-associated reactions were included when no corresponding gene was annotated in *P. putida*'s genome but physiological or experimental data supported the presence of the biochemical transformation being part of *P. putida*'s metabolism. Finally the reversibility was determined from primary literature data for each particular enzyme/reaction, if available. This literature search resulted in a first manually-curated reconstruction specific to *P. putida's *metabolism based on genome annotation and available biochemical evidence. However, this list is normally incomplete and will contain network gaps that may need to be filled depending on supporting evidence. This step requires manual effort again by searching the scientific literature for supporting information. If no *P. putida*-specific experimental evidence could be found for a transport reaction or biochemical transformation of a metabolite, no reaction or transporter was added to the network. Finally, the network capabilities were evaluated and compared with experimental data as described in Reed et al. [30]. Detailed lists of the genes, proteins, and reactions are contained in the Additional file 8, and the definitions of all metabolites and their abbreviations are found in the Additional file 9.

SimPheny (Genomatica Inc., San Diego, CA) software was used for the reconstruction and gap evaluation process.

### Conversion of the network reconstruction to a condition-specific model

The reconstructed metabolic network is often represented in a tabular format, listing all network reactions and metabolites in a human-readable manner along with confidence scores and comments (see Reed et al [30] for details). The conversion into a mathematical, or computer-readable format, can be done automatically by parsing the stoichiometric coefficients from the network reaction list (e.g. using the COBRA toolbox [92]). The mathematical format is called a stoichiometric matrix, or S-matrix, where the rows correspond to the network metabolites and the columns represent the network reactions. For each reaction, the stoichiometric coefficients of the substrates are listed with a minus sign in the corresponding cell of the matrix, while the product coefficients are positive numbers, by definition. The resulting size of the S-matrix is m × n, where m is the number of metabolites and n the number of network reactions. Mathematically, the S-matrix is a linear transformation of the flux vector *v *= (*v*_1_, *v*_2_,.., *v*_*n*_) to a vector of time derivatives of the concentration vector *x *= (*x*_1_, *x*_2_,.., *x*_*m*_) as $\frac{dx}{dt}=S\cdot v$. At steady-state, the change in concentration as a function of time is zero; hence, it follows: $\frac{dx}{dt}=S\cdot v$ = 0. The set of possible flux vectors v that satisfy this equality constraint might be subject to further constraints by defining *v*_*i*,min_≤ *v*_*i *_≤ *v*_*i*,max _for reaction *i*. In fact, for every irreversible network reaction *i*, the lower bound was defined as *v*_*i*,min _≥ 0 and the upper bound was defined as *v*_*i*,max _≥ 0.

Exchange reactions, which supply the network with nutrients or remove secretion products from the medium, were defined for all known medium components (see Additional file 9 for details). The uptake of a substrate by the network was defined by a flux rate *v*_*i *_< 0 and secretion of a by-product was defined to be *v*_*i *_> 0 for every exchange reaction *i*. An exchange reaction is represented in the reaction is as follows: e.g. D-glucose exchange: Ex_glc-D: 1 glc-D →. Note that this exchange reaction is unbalanced. Exchange (uptake) reactions define the presence of media components as if one would add metabolites into an *in silico *flask.

Finally, the application of constraints corresponding to different environmental conditions (e.g. minimal growth medium) or different genetic background (e.g. enzyme-deficient mutant) allow the transition from metabolic network reconstruction to condition-specific model. Note that the metabolic network reconstruction is unique to the target organism (and defined by its genome) while it can give rise to many different models by applying condition-specific constraints. All flux rates, *v*_*i*_, except biomass formation, are given in mmol/gDW/h.

### Biomass function

It is generally assumed that the objective of living organisms is to divide and proliferate. Subsequently, many metabolic network reconstructions have a so-called biomass function, in which all known metabolic precursors of cellular biomass are gathered (e.g. amino acids, nucleotides, phospholipids, vitamins, cofactors, energetic requirements etc.) [36-39]. Since no detailed studies about *P. putida's *biomass composition are available, the biomass composition from *E. coli *[55,93] was used as a template for *i*JN746's biomass function. However, data from *P. putida *were added, (e.g. membrane phospholipid composition [94]), when available. The detailed calculation of the biomass composition is provided in the Additional file 10.

### *in silico *medium composition

Aerobic growth was modeled in two different culture media: *in silico *M9 minimal medium (*i*M9) and *in silico *Luria-Bertani medium (*i*LB) [37]. For *i*M9 simulation, and according to the well described M9 minimal medium [90], the following external metabolites, CO_2_, Co_2 _^+^, Fe_2 _^+^, H^+^, H_2_O, Na_2 _^+^, Ni_2 _^+^, NH_4_, P_i _and SO_4 _were allowed to enter and leave the network by setting the constraints on the corresponding exchange reactions (*i*) to *v*_*i*,min_≥ -10^6 ^mmol/gDW/h and to *v*_*i*,max_≤ 10^6 ^mmol/gDW/h. The uptake rate for each carbon source was constrained to *v*_*i*,min_≥ -10 mmol/gDW/h and *v*_*i*,max_≤ 0 mmol/gDW/h. The oxygen uptake rate (OUR) was limited to *v*_*i*,min_≥ -18.5 mmol/gDW/h (based on *E. coli *data [95]), if not noted differently. In each individual simulation, all other external metabolites were only allowed to leave the system by constraining their exchange fluxes *i *between *v*_*i*,min_≥ 0 and *v*_*i*,max_≥ 10^6 ^mmol/gDW/h. The *i*LB medium was based on the published analysis of yeast extract and tryptone provided by the corresponding manufactures, and the *i*LB simulations were performed according previously published methods [37].

### Phenotypic phase-plane analysis

Phenotypic phase-plane analysis (PhPP) was carried out using SimPheny (Genomatica Inc., San Diego, CA). The underlying algorithm was described elsewhere [96,97]. The simulation was carried out using *i*M9 minimal medium (as described above) and setting the bounds of toluene uptake between *v*_*i*,min_≥ -11.9 mmol/gDW/h (based on measurement by [26] and *v*_*i*,max_≤ 0 mmol/gDW/h; and of oxygen between *v*_*i*,min_≥ -160 mmol/gDW/h and *v*_*i*,max_≤ 0 mmol/gDW/h. The step size was chosen to be 35.

### Reduced Cost

Reduced cost is a parameter of linear programming (LP) problems which is associated with each network reaction (*v*_*i*_) and represents the amount by which the objective function (e.g. growth rate) could be increased when the flux rate through this reaction was increased by a single unit [77]. Reduced cost is often used to analyze the obtained optimal solution and evaluate alternate solutions from the original solution [77]. In this study, we analyzed the reduced costs associated with uptake reactions to identify candidate reactions through which an increased flux would result in a higher growth rate (under the chosen simulation condition). The growth condition was *i*M9 medium with toluene as carbon source. The constraints were set as described above and linear programming was employed to solve the optimization problem (maximizing growth).

### Gene essentiality and auxotrophy

In order to determine the effect of a single gene deletion, all the reactions associated with each gene in *i*JN746 were individually "deleted" by setting the flux to 0 and optimizing for the biomass function [32]. A lethal deletion was defined if no positive flux value for the biomass function could be obtained for the given mutant *in silico *strain and medium. The simulations were performed using i) *i*LB rich medium for general gene essentially experiment and ii) glucose-iM9 minimal medium for auxotrophy experiments (See above). The glucose uptake rate was fixed to *v*_*i*,min _= *v*_*i*,max _= -6.3 mmol/gDW/h in the latter study. OUR was set to be *v*_*i*,min_≥ -18.5 mmol/gDW/h in both cases.

### msc-PHA production

The msc-PHA production from each possible carbon source (Table 2) in *i*M9 medium was determined by setting the growth rate to *v*_*growth*,min _= *v*_*growth*,max _0.2 gDW/gDW/h. The lower bound of each carbon uptake reaction was set to *v*_*i*,min_≥ -10 mmol/gDW/h and the upper bound was set to be *v*_*i*,max_≤ 0 mmol/gDW/h. The lower bound of the oxygen uptake rate was set to *v*_*i*,min_≥ -20 mmol/gDW/h for all simulations. In *i*JN746, six types of msc-PHAs are defined as well as msc-PHA compounds consisting of four different carbon chains [see Figure 5 and Additional file 7]. The corresponding demand functions were used as objective functions independently for the optimization problem. The resulting msc-PHA production rates were scaled by the number of carbons of the corresponding carbon sources to facilitate a yield comparison.

### Software

All computational simulations were performed using Matlab (The MathWorks Inc., Natick, MA) if not stated otherwise. TomLab (Tomlab Optimization Inc., San Diego, CA) was used as linear programming solver. Optimization formulations and the gene deletion studies employed the Matlab-based COBRA toolbox [92].

## Authors' contributions

All authors conceived the study. JN carried out the reconstruction of *Pseudomonas putida *KT2440. JN and IT performed the analyses. JN, IT, BOP designed the study and wrote manuscript. All authors read and approved the final manuscript.

## Supplementary Material

Additional file 1 **Table S1**. Carbon, nitrogen, and sulfur sources, which enabled growth of *i*JN746. Click here for file

Additional file 2 **Table S2**. Essentials genes predicted correctly in *i*JN746 compared with experimental data of *P. aeruginosa*. Click here for file

Additional file 3 **Figure S1**. Schematic representation of *in silico *gene essentiality in *i*JN746 (*i*LB medium) compared experimental data of gene essentiality in *P. aeruginosa *[83]. Click here for file

Additional file 4 **Table S3**. False-positive essential genes in *i*JN746 when compared with *P. aeruginosa*'s experimental data [83]. Click here for file

Additional file 5 **Table S4**. False-negative essential genes in *i*JN746. Genes that were not predicted to be essential in *i*JN746 but were reported as essential genes in *P. aeruginosa *[83]. Click here for file

Additional file 6 **Table S5**. Predicted essential genes in Glucose-*i*M9 minimal medium. Not shown are genes that were also predicted to be essential in *i*LB rich medium. Click here for file

Additional file 7 **Table S6**. PHA polymer composition found in different *Pseudomonas *strains sorted by carbon sources. Click here for file

Additional file 8 **Table S7**. List of metabolites in *i*JN746. The file contains a detail list of metabolites present in the metabolic reconstruction. The molecular formulae, the charge as well as the KeggID are shown. Click here for file

Additional file 9 **Table S8**. List of the reactions contain in iJN746. The file details the reactions account in the metabolic reconstruction. The official name, the equation of the reaction, the subsystem, the EC number and de GPR association is shown. Click here for file

Additional file 10 **Table S9**. List of biomass components in *i*JN746. This file contains the complete list of compounds which are part of *Pseudomonas putida *biomass. Click here for file

## References

[B1] Clarke P, Richmond MH (1975). Genetics and Biochemistry of Pseudomonas.

[B2] Clarke P (1982). The metabolic versatility of pseudomonads. Antonie Van Leeuwenhoek.

[B3] Franklin FC, Bagdasarian M, Bagdasarian MM, Timmis K (1981). Molecular and functional analysis of the TOL plasmid pWWO from Pseudomonas putida and cloning of genes for the entire regulated aromatic ring meta cleavage pathway. Proc Natl Acad Sci USA.

[B4] Bayley SA, Duggleby CJ, Worsey MJ, Williams PA, Hardy KG, Broda aP (1977). Two modes of loss of the Tol function from Pseudomonas putida mt-2. Mol Gen Genet.

[B5] Mermod N, Harayama S, Timmis K (1986). New route to bacterial production of indigo. Bio/Technology.

[B6] Ramos J, Wasserfallen A, Rose K, Timmis K (1987). Redesigning metabolic routes: manipulation of TOL plasmid pathway for catabolism of alkylbenzoates. Science.

[B7] Cases I, de Lorenzo V (1998). Expression systems and physiological control of promoter activity in bacteria. Curr Opin Microbiol.

[B8] Gilbert ES, Walker AW, Keasling J (2003). A constructed microbial consortium for biodegradation of the organophosphorus insecticide parathion. Appl Microbiol Biotechnol.

[B9] Timmis KN, Steffan RJ, Unterman R (1994). Designing microorganisms for the treatment of toxic wastes. Annu Rev Microbiol.

[B10] Dejonghe W, Boon N, Seghers D, Top EM, Verstraete W (2001). Bioaugmentation of soils by increasing microbial richness: missing links. Environ Microbiol.

[B11] Galán B, Díaz E, García JL (2000). Enhancing desulphurization by engineering a flavin reductase-encoding gene cassette in recombinant biocatalysts. Environ Microbiol.

[B12] Zeyer J, Lehrbach PR, Timmis KN (1985). Use of cloned genes of Pseudomonas TOL plasmid to effect biotransformation of benzoates to cis-dihydrodiols and catechols by Escherichia coli cells. Appl Environ Microbiol.

[B13] Wubbolts MG, Timmis KN (1990). Biotransformation of substituted benzoates to the corresponding cis-diols by an engineered strain of Pseudomonas oleovorans producing the TOL plasmid-specified enzyme toluate-1,2-dioxygenase. Appl Environ Microbiol.

[B14] Schmid A, Dordick JS, Hauer B, Kiener A, Wubbolts M, Witholt B (2001). Industrial biocatalysis today and tomorrow. Nature.

[B15] Olivera ER, Carnicero D, Jodra R, Minambres B, Garcia B, Abraham GA, Gallardo A, Roman JS, Garcia JL, Naharro G (2001). Genetically engineered Pseudomonas: a factory of new bioplastics with broad applications. Environmental Microbiology.

[B16] Ouyang SP, Luo RC, Chen SS, Liu Q, Chung A, Wu Q, Chen GQ (2007). Production of Polyhydroxyalkanoates with High 3-Hydroxydodecanoate Monomer Content by fadB and fadA Knockout Mutant of Pseudomonas putida KT2442. Biomacromolecules.

[B17] Huijberts GN, Eggink G, de Waard P, Huisman GW, Witholt B (1992). Pseudomonas putida KT2442 cultivated on glucose accumulates poly(3-hydroxyalkanoates) consisting of saturated and unsaturated monomers. Appl Environ Microbiol.

[B18] O'Sullivan DJ, O'Gara F (1992). Traits of fluorescent Pseudomonas spp. involved in suppression of plant root pathogens. Microbiol Rev.

[B19] Walsh UF, Morrissey JP, O'Gara F (2001). Pseudomonas for biocontrol of phytopathogens: from functional genomics to commercial exploitation. Curr Opin Biotechnol.

[B20] Nelson KE, Weinel C, Paulsen IT, Dodson RJ, Hilbert H, Martins dos Santos VAP, Fouts DE, Gill SR, Pop M, Holmes M (2002). Complete genome sequence and comparative analysis of the metabolically versatile Pseudomonas putida KT2440. Environmental Microbiology.

[B21] Ramos JL (2004). Pseudomonas.

[B22] Yuste L, Hervas AB, Canosa I, Tobes R, Jimenez JI, Nogales J, Perez-Perez MM, Santero E, Diaz E, Ramos J-L (2006). Growth phase-dependent expression of the Pseudomonas putida KT2440 transcriptional machinery analysed with a genome-wide DNA microarray. Environmental Microbiology.

[B23] Dominguez-Cuevas P, Gonzalez-Pastor J-E, Marques S, Ramos J-L, de Lorenzo V (2006). Transcriptional Tradeoff between Metabolic and Stress-response Programs in Pseudomonas putida KT2440 Cells Exposed to Toluene. J Biol Chem.

[B24] Kim Young Hwan, Sung-Ho Kun Cho, Young Yun Jin, Kyung-Hoon Kim, Shin Kwon Jong, YSI Kim (2006). Analysis of aromatic catabolic pathways in Pseudomonas putida KT 2440 using a combined proteomic approach: 2-DE/MS and cleavable isotope-coded affinity tag analysis. PROTEOMICS.

[B25] del Castillo T, Ramos JL, Rodriguez-Herva JJ, Fuhrer T, Sauer U, Duque E (2007). Convergent Peripheral Pathways Catalyze Initial Glucose Catabolism in Pseudomonas putida: Genomic and Flux Analysis. J Bacteriol.

[B26] del Castillo T, Ramos JL (2007). Simultaneous Catabolite Repression between Glucose and Toluene Metabolism in Pseudomonas putida Is Channeled through Different Signaling Pathways. J Bacteriol.

[B27] Jimenez JI, Minambres B, Garcia JL, Diaz E (2002). Genomic analysis of the aromatic catabolic pathways from Pseudomonas putida KT2440. Environmental Microbiology.

[B28] dos Santos VAPM, Heim S, Moore ERB, Stratz M, Timmis KN (2004). Insights into the genomic basis of niche specificity of Pseudomonas putida KT2440. Environmental Microbiology.

[B29] Palsson BØ (2004). In silico biotechnology. Era of reconstruction and interrogation. Curr Opin Biotechnol.

[B30] Reed JL, Famili I, Thiele I, Palsson BO (2006). Towards multidimensional genome annotation. Nat Rev Genet.

[B31] Palsson BO (2004). Two-dimensional annotation of genomes. Nat Biotechnol.

[B32] Price ND, Reed JL, Palsson BO (2004). Genome-scale models of microbial cells: evaluating the consequences of constraints. Nat Rev Micro.

[B33] Becker SA, Feist AM, Mo ML, Hannum G, Palsson BO, Herrgard MJ (2007). Quantitative Prediction of Cellular Metabolism with Constraint-based Models: The COBRA Toolbox. Nat Protoc.

[B34] Price ND, Reed JL, Palsson BO (2004). Genome-scale models of microbial cells: evaluating the consequences of constraints. Nat Rev Microbiol.

[B35] Feist AM, Scholten JCM, Palsson BO, Brockman FJ, Ideker T (2006). Modeling methanogenesis with a genome-scale metabolic reconstruction of Methanosarcina barkeri. Mol Syst Biol.

[B36] Feist AM, Henry CS, Reed JL, Krummenacker M, Joyce AR, Karp PD, Broadbelt LJ, Hatzimanikatis V, Palsson BO (2007). A genome-scale metabolic reconstruction for Escherichia coli K-12 MG1655 that accounts for 1260 ORFs and thermodynamic information. Mol Syst Biol.

[B37] Oh Y-K, Palsson BO, Park SM, Schilling CH, Mahadevan R (2007). Genome-scale Reconstruction of Metabolic Network in Bacillus subtilis Based on High-throughput Phenotyping and Gene Essentiality Data. J Biol Chem.

[B38] Thiele I, Vo TD, Price ND, Palsson B (2005). An Expanded Metabolic Reconstruction of Helicobacter pylori (*i*IT341 GSM/GPR): An *in silico *genome-scale characterization of single and double deletion mutants. J Bacteriol.

[B39] Jamshidi N, Palsson B (2007). Investigating the metabolic capabilities of Mycobacterium tuberculosis H37Rv using the in silico strain iNJ661 and proposing alternative drug targets. BMC Systems Biology.

[B40] Beste DJ, Hooper T, Stewart G, Bonde B, Avignone-Rossa C, Bushell ME, Wheeler P, Klamt S, Kierzek AM, McFadden J (2007). GSMN-TB: a web-based genome-scale network model of Mycobacterium tuberculosis metabolism. Genome Biol.

[B41] Becker SA, Palsson BO (2005). Genome-scale reconstruction of the metabolic network in Staphylococcus aureus N315: an initial draft to the two-dimensional annotation. BMC Microbiol.

[B42] Heinemann M, Kummel A, Ruinatscha R, Panke S (2005). In silico genome-scale reconstruction and validation of the Staphylococcus aureus metabolic network. Biotechnol Bioeng.

[B43] Oliveira AP, Nielsen J, Forster J (2005). Modeling Lactococcus lactis using a genome-scale flux model. BMC Microbiol.

[B44] Duarte NC, Becker SA, Jamshidi N, Thiele I, Mo ML, Vo TD, Srivas R, Palsson BO (2007). Global reconstruction of the human metabolic network based on genomic and bibliomic data. Proceedings of the National Academy of Sciences.

[B45] Reed JL, Patel TR, Chen KH, Joyce AR, Applebee MK, Herring CD, Bui OT, Knight EM, Fong SS, Palsson BO (2006). Systems approach to refining genome annotation. Proceedings of the National Academy of Sciences.

[B46] Ibarra RU, Edwards JS, Palsson BO (2002). *Escherichia coli *K-12 undergoes adaptive evolution to achieve *in silico *predicted optimal growth. Nature.

[B47] Joyce AR, Fong SS, Palsson BO (2004). Adaptive Evolution of *E. coli *on Either Lactate or Glycerol Leads to Convergent, Generalist Phenotypes. International E Coli Alliance Second Annual Meeting: 2004; Banff, Alberta.

[B48] Fong SS, Palsson BO (2004). Metabolic gene deletion strains of Escherichia coli evolve to computationally predicted growth phenotypes. Nature Genetics.

[B49] Park JH, Lee KH, Kim TY, Lee SY (2007). Metabolic engineering of Escherichia coli for the production of L-valine based on transcriptome analysis and in silico gene knockout simulation. Proceedings of the National Academy of Sciences.

[B50] Thiele I, Price ND, Vo TD, Palsson BO (2005). Candidate metabolic network states in human mitochondria: Impact of diabetes, ischemia, and diet. J Biol Chem.

[B51] Ravasz E, Somera AL, Mongru DA, Oltvai ZN, Barabasi AL (2002). Hierarchical organization of modularity in metabolic networks. Science.

[B52] Barabasi AL, Oltvai ZN (2004). Network biology: understanding the cell's functional organization. Nature reviews.

[B53] Almaas E, Kovacs B, Vicsek T, Oltvai ZN, Barabasi AL (2004). Global organization of metabolic fluxes in the bacterium *Escherichia coli*. Nature.

[B54] Feist AM, Palsson BO (2008). Metabolic Flux Balancing: Basic concepts, Scientific and Practical Use – 13 Years Later. Nat Biotechnol.

[B55] Reed JL, Vo TD, Schilling CH, Palsson BO (2003). An expanded genome-scale model of *Escherichia coli *K-12 (*i*JR904 GSM/GPR). Genome Biology.

[B56] Varma A, Palsson BO (1994). Stoichiometric flux balance models quantitatively predict growth and metabolic by-product secretion in wild-type Escherichia coli W3110. Appl Environ Microbiol.

[B57] Kanehisa M, Goto S, Hattori M, Aoki-Kinoshita KF, Itoh M, Kawashima S, Katayama T, Araki M, Hirakawa M (2006). From genomics to chemical genomics: new developments in KEGG. Nucl Acids Res.

[B58] Romero P, Karp P (2003). PseudoCyc, A Pathway-Genome Database for Pseudomonas aeruginosa. Journal of Molecular Microbiology and Biotechnology.

[B59] Choi C, Munch R, Leupold S, Klein J, Siegel I, Thielen B, Benkert B, Kucklick M, Schobert M, Barthelmes J (2007). SYSTOMONAS – an integrated database for systems biology analysis of Pseudomonas. Nucl Acids Res.

[B60] Revelles O, Wittich R-M, Ramos JL (2007). Identification of the Initial Steps in D-Lysine Catabolism in Pseudomonas putida. J Bacteriol.

[B61] Huijberts GN, de Rijk TC, de Waard P, Eggink G (1994). 13C nuclear magnetic resonance studies of Pseudomonas putida fatty acid metabolic routes involved in poly(3-hydroxyalkanoate) synthesis. J Bacteriol.

[B62] Hazer B, Steinbüchel A (2007). Increased diversification of polyhydroxyalkanoates by modification reactions for industrial and medical applications. Appl Microbiol Biotechnol.

[B63] Madison LL, Huisman GW (1999). Metabolic Engineering of Poly(3-Hydroxyalkanoates): From DNA to Plastic. Microbiol Mol Biol Rev.

[B64] Oberhardt MA, Puchalka J, Fryer KE, Martins dos Santos VAP, Papin JA (2008). Genome-Scale Metabolic Network Analysis of the Opportunistic Pathogen Pseudomonas aeruginosa PAO1. J Bacteriol.

[B65] Janssen P, Goldovsky L, Kunin V, Darzentas N, Ouzounis CA (2005). Genome coverage, literally speaking. The challenge of annotating 200 genomes with 4 million publications. EMBO Rep.

[B66] Ryan PR, Delhaize E, Jones DL (2001). Function and mechanism Of Organic anion exudation from plant roots. Annual Review of Plant Physiology and Plant Molecular Biology.

[B67] Espinosa-Urgel M, Ramos J-L (2001). Expression of a Pseudomonas putida Aminotransferase Involved in Lysine Catabolism Is Induced in the Rhizosphere. Appl Environ Microbiol.

[B68] Stanier RY, Palleroni N, Doudoroff M (1966). The aerobic pseudomonads: a taxonomic study. J Gen Microbiol.

[B69] Galvao TC, de Lorenzo V, Canovas D (2006). Uncoupling of choline-O-sulphate utilization from osmoprotection in Pseudomonas putida. Molecular Microbiology.

[B70] Vicente M, Canovas JL (1973). Glucolysis in Pseudomonas putida: Physiological Role of Alternative Routes from the Analysis of Defective Mutants. J Bacteriol.

[B71] Reed JL, Famili I, Thiele I, Palsson BO (2006). Towards multidimensional genome annotation. Nat Rev Genet.

[B72] Worsey MJ, Williams PA (1975). Metabolism of toluene and xylenes by Pseudomonas (putida (arvilla) mt-2: evidence for a new function of the TOL plasmid. J Bacteriol.

[B73] Assinder SJ, PA W (1990). The TOL plasmids: determinants of the catabolism of toluene and the xylenes. Adv Microb Physiol.

[B74] Harayama S, Rekik M, Wubbolts M, Rose K, Leppik RA, Timmis KN (1989). Characterization of five genes in the upper-pathway operon of TOL plasmid pWW0 from Pseudomonas putida and identification of the gene products. J Bacteriol.

[B75] Harayama S, Rekik M (1990). The meta cleavage operon of TOL degradative plasmid pWW0 comprises 13 genes. Mol Gen Genet.

[B76] Ramos JL, Marques S, Timmis KN (1997). Transcriptional control of the pseudomonas tol plasmid catabolic operons is achieved through an interplay of host factors and plasmid-encoded regulators. Annual Review of Microbiology.

[B77] Ramakrishna R, Edwards JS, McCulloch A, Palsson BO (2001). Flux-balance analysis of mitochondrial energy metabolism: consequences of systemic stoichiometric constraints. American journal of physiology.

[B78] Fischer E, Zamboni N, Sauer U (2004). High-throughput metabolic flux analysis based on gas chromatography-mass spectrometry derived 13C constraints. Anal Biochem.

[B79] Alagappan G, Cowan RM (2004). Effect of temperature and dissolved oxygen on the growth kinetics of Pseudomonas putida F1 growing on benzene and toluen. Chemosphere.

[B80] Denef VJ, Klappenbach JA, Patrauchan MA, Florizone C, Rodrigues JLM, Tsoi TV, Verstraete W, Eltis LD, Tiedje JM (2006). Genetic and Genomic Insights into the Role of Benzoate-Catabolic Pathway Redundancy in Burkholderia xenovorans LB400. Appl Environ Microbiol.

[B81] Fridovich I (1978). Superoxide radicals, superoxide dismutases and the aerobic lifestyle. Photochem Photobiol.

[B82] Jacobs MA, Alwood A, Thaipisuttikul I, Spencer D, Haugen E, Ernst S, Will O, Kaul R, Raymond C, Levy R (2003). Comprehensive transposon mutant library of Pseudomonas aeruginosa. Proceedings of the National Academy of Sciences.

[B83] Liberati NT, Urbach JM, Miyata S, Lee DG, Drenkard E, Wu G, Villanueva J, Wei T, Ausubel FM (2006). An ordered, nonredundant library of Pseudomonas aeruginosa strain PA14 transposon insertion mutants. Proceedings of the National Academy of Sciences.

[B84] Ward PG, de Roo G, O'Connor KE (2005). Accumulation of Polyhydroxyalkanoate from Styrene and Phenylacetic Acid by Pseudomonas putida CA-3. Appl Environ Microbiol.

[B85] Timm A, Steinbuchel A (1990). Formation of polyesters consisting of medium-chain-length 3-hydroxyalkanoic acids from gluconate by Pseudomonas aeruginosa and other fluorescent pseudomonads. Appl Environ Microbiol.

[B86] Burgard AP, Pharkya P, Maranas CD (2003). Optknock: a bilevel programming framework for identifying gene knockout strategies for microbial strain optimization. Biotechnol Bioeng.

[B87] Pharkya P, Burgard AP, Maranas CD (2004). OptStrain: a computational framework for redesign of microbial production systems. Genome Res.

[B88] Hua Q, Joyce AR, Fong SS, Palsson BO (2006). Metabolic analysis of adaptive evolution for in silico designed lactate-producing strains. Biotechnol Bioeng.

[B89] Lee SY, Lee DY, Kim TY (2005). Systems biotechnology for strain improvement. Trends Biotechnol.

[B90] Abril MA, Michan C, Timmis KN, Ramos JL (1989). Regulator and enzyme specificities of the TOL plasmid-encoded upper pathway for degradation of aromatic hydrocarbons and expansion of the substrate range of the pathway. J Bacteriol.

[B91] Fuhrer T, Fischer E, Sauer U (2005). Experimental Identification and Quantification of Glucose Metabolism in Seven Bacterial Species. J Bacteriol.

[B92] Becker SA, Feist AM, Mo ML, Hannum G, Palsson BO, Herrgard MJ (2007). Quantitative prediction of cellular metabolism with constraint-based models: the COBRA Toolbox. Nat Protocols.

[B93] Neidhardt FC, Ingraham JL, Schaechter M (1990). Physiology of the bacterial cell: a molecular approach.

[B94] Pinkart HC, White DC Lipids of pseudomonas.

[B95] Edwards JS, Ibarra RU, Palsson B (2001). In silico predictions of Escherichia coli metabolic capabilities are consistent with experimental data. Nat Biotechnol.

[B96] Schilling CH, Edwards JS, Letscher D, Palsson BO (2000). Combining pathway analysis with flux balance analysis for the comprehensive study of metabolic systems. Biotechnol Bioeng.

[B97] Edwards JS, Ibarra RU, Palsson BO (2001). *In silico *predictions of *Escherichia coli *metabolic capabilities are consistent with experimental data. Nat Biotechnol.

[B98] Riley M, Abe T, Arnaud MB, Berlyn MK, Blattner FR, Chaudhuri RR, Glasner JD, Horiuchi T, Keseler IM, Kosuge T (2006). *Escherichia coli *K-12: a cooperatively developed annotation snapshot-2005. Nucleic Acids Res.

[B99] Nogales J, Canales A, Jimenez-Barbero J, Garcia JL, Diaz E (2005). Molecular Characterization of the Gallate Dioxygenase from Pseudomonas putida KT2440: The prototype of a new subgroup of extradiol dioxygenases. J Biol Chem.

[B100] Fan CL, Miller DL, Rodwell VW (1972). Metabolism of Basic Amino Acids in Pseudomonas putida. Transport of lysine, ornithine, and arginine. J Biol Chem.

[B101] Vilchez S, Molina L, Ramos C, Ramos JL (2000). Proline Catabolism by Pseudomonas putida: Cloning, Characterization, and Expression of the put Genes in the Presence of Root Exudates. J Bacteriol.

[B102] Haywood GW, Anderson AJ, Ewing DF, Dawes EA (1990). Accumulation of a Polyhydroxyalkanoate Containing Primarily 3-Hydroxydecanoate from Simple Carbohydrate Substrates by Pseudomonas sp. Strain NCIMB 40135. Appl Environ Microbiol.

[B103] Huisman GW, de Leeuw O, Eggink G, Witholt B (1989). Synthesis of poly-3-hydroxyalkanoates is a common feature of fluorescent pseudomonads. Appl Environ Microbiol.

