# Supplementary material for: A genome-scale metabolic reconstruction of Pseudomonas putida KT2440: iJN746 as a cell factory
Source: BMC Syst Biol. 2008 Sep 16;2:79. doi: 10.1186/1752-0509-2-79 (PMC2569920; doi:10.1186/1752-0509-2-79)
Supplement: Additional file 1 — Table S1. Carbon, nitrogen, and sulfur sources, which enabled growth of iJN746. [file 1752-0509-2-79-S1.doc]

**Additional file 1: Table S1.** Carbon, nitrogen, and sulfur sources, which enabled growth of *i*JN746. Word file.

| **Compound** | **Carbon Source**  **(iJN746/*P.putida*)** | **Nitrogen Source**  **(iJN746/*P.putida*)** | **Sulfure Source**  **(iJN746/*P.putida*)** | **Reference** |
| --- | --- | --- | --- | --- |
| **2-Ketogluconate** | (+/+) | nd | nd | [68] |
| **Protocatechuate** | (+/+) | nd | nd | [27] |
| **Caffeate** | (+/+) | nd | nd | [27] |
| **Oxoadipate** | (+/+) | nd | nd | [68] |
| **4-Aminobutyrate** | (+/+) | (+/+) | nd | [68] |
| **4-Hydroxybenzoate** | (+/+) | nd | nd | [27] |
| **Acetate** | (+/+) | nd | nd | [68] |
| **α-Ketoglutarate** | (+/+) | nd | nd | [68] |
| **L-Alanine** | (-/+) | (-/+) | nd | [68] |
| **L-Arginine** | (+/+) | (+/+) | nd | [68] |
| **L-Aspartate** | (+/+) | (+/+) | nd | [68] |
| **Benzoate** | (+/+) | nd | nd | [27] |
| **Catechol** | (+/+) | nd | nd | [27] |
| **Choline** | (+/+) | (+/+) | nd | [69] |
| **Sulfate choline** | (+/+) | (+/+) | (+/+) | [69] |
| **Citrate** | (+/+) | nd | nd | [68] |
| **Coniferyl alcohol** | (+/+) | nd | nd | [27] |
| **L-Cysteine** | (-/-) | (+/+) | (+/+) | [21] |
| **Decanoate** | (+/+) | nd | nd | [17] |
| **Dodecanoate** | (+/+) | nd | nd | [17] |
| **Ethanesulfonate** | nd | nd | (+/+) | [21] |
| **Ferulate** | (+/+) | nd | nd | [27] |
| **Fructose** | (+/+) | nd | nd | [68] |
| **Fumarate** | (+/+) | nd | nd | [68] |
| **Gallate** | (+/+) | nd | nd | [99] |
| **Glucose** | (+/+) | nd | nd | [68] |
| **Gluconate** | (+/+) | nd | nd | [68] |
| **L-Glutamate** | (+/+) | (+/+) | nd | [68] |
| **L-Glycine** | (+/+) | (+/+) | nd | [68] |
| **Glyceraldehyde** | (+/+) | nd | nd | [68] |
| **Glycine Betaine** | (+/+) | (+/+) | nd | [69] |
| **Glycerate** | (+/+) | nd | nd | [68] |
| **Glycolate** | (+/+) | nd | nd | [68] |
| **Hexadecanoate** | (+/+) | nd | nd | [17] |
| **L-Histidine** | (+/+) | (+/+) | nd | [68] |
| **Hexanoate** | (+/+) | nd | nd | [17] |
| **Isocitrate** | (+/+) | nd | nd | [68] |
| **L-Isoleucine** | (+/+) | (+/+) | nd | [68] |
| **Isethionic acid** | nd | nd | (+/+) | [21] |
| **D-Lactate** | (+/+) | nd | nd | [68] |
| **L-Lactate** | (+/+) | nd | nd | [68] |
| **L-Leucine** | (+/+) | (+/+) | nd | [68] |
| **L-Lysine** | (+/+) | (+/+) | nd | [60] |
| **m-Xylene** | (+/+) | nd | nd | [72] |
| **L-Malate** | (+/+) | nd | nd | [68] |
| **L-Metionine** | (-/-) | (+/+) | (+/+) | [21] |
| **Methanesulfonate** | nd | nd | (+/+) | [21] |
| **Nicotinate** | (+/+) | (+/+) | nd | [27] |
| **Amonium** | nd | (+/+) | nd | [21] |
| **Nitrite** | nd | (+/+) | nd | [21] |
| **Octanoate** | (+/+) | nd | nd | [61] |
| **Ornitine** | (+/+) | (+/+) | nd | [100] |
| **p-Xylene** | (+/+) | nd | nd | [72] |
| **Phenylacetate** | (+/+) | nd | nd | [27] |
| **Penthanesulfonate** | nd | nd | (+/+) | [21] |
| **L-Phenylalanine** | (+/+) | (+/+) | nd | [27] |
| **L-Proline** | (+/+) | (+/+) | nd | [101] |
| **Putrescine** | (-/+) | (-/+) | nd | [68] |
| **Quinate** | (+/+) | nd | nd | [27] |
| **D-Ribose** | (+/+) | nd | nd | [68] |
| **L-Serine** | (+/+) | (+/+) | nd | [68] |
| **Sulfate** | nd | nd | (+/+) | [21] |
| **Spermidine** | (-/+) | (-/+) | nd | [68] |
| **Succinate** | (+/+) | nd | nd | [68] |
| **Taurine** | nd | nd | (+/+) | [21] |
| **p-Coumarate** | (+/+) | nd | nd | [27] |
| **L-Treonine** | (+/+) | (+/+) | nd | [68] |
| **Toluene** | (+/+) | nd | nd | [72] |
| **Tiosulphate** | nd | nd | (+/+) | [21] |
| **Tetradecanoate** | (+/+) | nd | nd | [61] |
| **L-Tyrosine** | (+/+) | (+/+) | nd | [27] |
| **L-Valine** | (+/+) | (+/+) | nd | [68] |
| **Vanilline** | (+/+) | nd | nd | [27] |
| **Vanilate** | (+/+) | nd | nd | [27] |

*In silico* growth capabilities were compared with experimental data from *P. putida*. (+) growth, (-) no growth and (nd) not determined
