# Supplementary material for: A genome-scale metabolic reconstruction of Pseudomonas putida KT2440: iJN746 as a cell factory
Source: BMC Syst Biol. 2008 Sep 16;2:79. doi: 10.1186/1752-0509-2-79 (PMC2569920; doi:10.1186/1752-0509-2-79)
Supplement: Additional file 2 — Table S2. Essentials genes predicted correctly in iJN746 compared with experimental data of P. aeruginosa. [file 1752-0509-2-79-S2.doc]

**Additional file 2: Table S2.** Essentials genes predicted correctly in *i*JN746 compared with experimental data of *P. aeruginosa.* Word file.

| **KT2440**  **gene** | **Gene name** | **PA14**  **orthologue** | **PA01**  **orthologue** | **Functional class** |
| --- | --- | --- | --- | --- |
| **PP_5046** | *glnA* | PA14_67600 | PA5119 | Amino acid biosynthesis and metabolism |
| **PP_5079** | *aroK* | PA14_66610 | PA5039 | Amino acid biosynthesis and metabolism |
| **PP_1237** | *dapA* | PA14_51270 | PA1010 | Amino acid biosynthesis and metabolism |
| **PP_1530** | *dapD* | PA14_16950 | PA3666 | Amino acid biosynthesis and metabolism |
| **PP_4725** | *dapB* | PA14_62940 | PA4759 | Amino acid biosynthesis and metabolism |
| **PP_0606** | *ispH* | PA14_60330 | PA4557 | Antibiotic resistance and susceptibility |
| **PP_0392** | *folB* | PA14_07590 | PA0582 | Biosynthesis of cofactors, prosthetic groups and carriers |
| **PP_0438** | *-* | PA14_08630 | PA4279 | Biosynthesis of cofactors, prosthetic groups and carriers |
| **PP_0527** | *dxs* | PA14_11550 | PA4044 | Biosynthesis of cofactors, prosthetic groups and carriers |
| **PP_0631** | *coaE1* | PA14_58780 | PA4529 | Biosynthesis of cofactors, prosthetic groups and carriers |
| **PP_0723** | *ispE* | PA14_61750 | PA4669 | Biosynthesis of cofactors, prosthetic groups and carriers |
| **PP_0732** | *hemA* | PA14_61710 | PA4666 | Biosynthesis of cofactors, prosthetic groups and carriers |
| **PP_0744** | *hemH* | PA14_61580 | PA4655 | Biosynthesis of cofactors, prosthetic groups and carriers |
| **PP_1597** | *dxr* | PA14_17130 | PA3650 | Biosynthesis of cofactors, prosthetic groups and carriers |
| **PP_4717** | *folP* | PA14_62850 | PA4750 | Biosynthesis of cofactors, prosthetic groups and carriers |
| **PP_5074** | *hemE* | PA14_66550 | PA5034 | Biosynthesis of cofactors, prosthetic groups and carriers |
| **PP_5285** | *coaBC* | PA14_70240 | PA5320 | Biosynthesis of cofactors, prosthetic groups and carriers |
| **PP_1337** | *murG* | PA14_57340 | PA4412 | Carbon compound catabolism |
| **PP_1338** | *murC* | PA14_57330 | PA4411 | Carbon compound catabolism |
| **PP_1332** | *murE* | PA14_57410 | PA4417 | Cell wall / LPS / capsule |
| **PP_1333** | *murF* | PA14_57390 | PA4416 | Cell wall / LPS / capsule |
| **PP_1334** | *mraY* | PA14_57380 | PA4415 | Cell wall / LPS / capsule |
| **PP_1904** | *murB* | PA14_25550 | PA2977 | Cell wall / LPS / capsule |
| **PP_0186** | *hemC* | PA14_69450 | PA5260 | Central intermediary metabolism |
| **PP_0187** | *hemD* | PA14_69440 | PA5259 | Central intermediary metabolism |
| **PP_5123** | *coaD* | PA14_04760 | PA0363 | Central intermediary metabolism |
| **PP_5132** | *folA* | PA14_04580 | PA0350 | Central intermediary metabolism |
| **PP_5409** | *glmS* | PA14_73170 | PA5549 | Central intermediary metabolism |
| **PP_0558** | *accC-1* | PA14_64110 | PA4848 | Fatty acid and phospholipid metabolism |
| **PP_0559** | *accB* | PA14_64100 | PA4847 | Fatty acid and phospholipid metabolism |
| **PP_0853** | *ispG* | PA14_14880 | PA3803 | Fatty acid and phospholipid metabolism |
| **PP_1596** | *cdsA* | PA14_17120 | PA3651 | Fatty acid and phospholipid metabolism |
| **PP_1607** | *accA* | PA14_17270 | PA3639 | Fatty acid and phospholipid metabolism |
| **PP_1914** | *fabG* | PA14_25660 | PA2967 | Fatty acid and phospholipid metabolism |
| **PP_1996** | *accD* | PA14_23860 | PA3112 | Fatty acid and phospholipid metabolism |
| **PP_4097** | *pgsA* | PA14_30670 | PA2584 | Fatty acid and phospholipid metabolism |
| **PP_4174** | *fabA* | PA14_43680 | PA1610 | Fatty acid and phospholipid metabolism |
| **PP_4908** | *psd* | PA14_65500 | PA4957 | Fatty acid and phospholipid metabolism |
| **PP_1031** | *guaB* | PA14_15310 | PA3770 | Nucleotide biosynthesis and metabolism |
| **PP_1177** | *nrdB* | PA14_49470 | PA1155 | Nucleotide biosynthesis and metabolism |
| **PP_1179** | *nrdA* | PA14_49460 | PA1156 | Nucleotide biosynthesis and metabolism |
| **PP_1610** | *pyrG* | PA14_17290 | PA3637 | Nucleotide biosynthesis and metabolism |
| **PP_1771** | *cmk* | PA14_23320 | PA3163 | Nucleotide biosynthesis and metabolism |
| **PP_3363** | *tmk* | PA14_25740 | PA2962 | Nucleotide biosynthesis and metabolism |
| **PP_4016** | *purB* | PA14_30110 | PA2629 | Nucleotide biosynthesis and metabolism |
| **PP_5141** | *thyA* | PA14_04480 | PA0342 | Nucleotide biosynthesis and metabolism |
| **PP_5286** | *dut* | PA14_70260 | PA5321 | Nucleotide biosynthesis and metabolism |
| **PP_1977** | *gltX* | PA14_23560 | PA3134 | Translation, post-translational modification, degradation |
