# Supplementary material for: A genome-scale metabolic reconstruction of Pseudomonas putida KT2440: iJN746 as a cell factory
Source: BMC Syst Biol. 2008 Sep 16;2:79. doi: 10.1186/1752-0509-2-79 (PMC2569920; doi:10.1186/1752-0509-2-79)
Supplement: Additional file 3 — Figure S1. Schematic representation of in silico gene essentiality in iJN746 (iLB medium) compared experimental data of gene essentiality in P. aeruginosa [83]. [file 1752-0509-2-79-S3.doc]

**Additional file 3: Figure S1.** Schematic representation of *in silico* gene essentiality in *i*JN746 (*i*LB medium) compared experimental data of gene essentiality in *P.aeruginosa* [83].

**Figure S1.** Schematic representation of *in silico* gene essentiality in *i*JN746 under *i*LB medium conditions. A total of 92 metabolic genes were predicted to be essential (*i*JN746PEG) in *i*LB mediun (red). 48 of these genes were correctly predicted when compared to the 335 essential genes found experimentally in *P.aeruginosa* (blue) [1]. The remaining 44 genes were only found to be essential in *i*JN746. Note that a significant number of these 335 essential genes found in *P. aeruginosa* were not metabolic. The predicted non-essential genes by *i*JN746 (*i*JN746NPEG) are shown in yellow. Only 37 of these genes were reported to be essential in *P. aeruginosa* (See text).
