# Supplementary material for: A genome-scale metabolic reconstruction of Pseudomonas putida KT2440: iJN746 as a cell factory
Source: BMC Syst Biol. 2008 Sep 16;2:79. doi: 10.1186/1752-0509-2-79 (PMC2569920; doi:10.1186/1752-0509-2-79)
Supplement: Additional file 4 — Table S3. False-positive essential genes in iJN746 when compared with P. aeruginosa's experimental data [83]. [file 1752-0509-2-79-S4.doc]

**Additional file 4: Table S3.** False-positive essential genes in *i*JN746 when compared with *P. aeruginosa*’s experimental data [83]. Word file.

| **KT2440 gene** | **Gene name** | **PA14**  **orthologo** | **PA01**  **orthologo** | **Functional Class** |
| --- | --- | --- | --- | --- |
| **PP_1525** | *dapE* | PA14_49380 | PA1162 | Amino acid biosynthesis and metabolism |
| **PP_1588** | *dapC* | PA14_17030 | PA3659 | Amino acid biosynthesis and metabolism |
| **PP_1750** | *asnB* | PA14_19370 | PA3459 | Amino acid biosynthesis and metabolism |
| **PP_1770** | *tyrA* | PA14_23310 | PA3164 | Amino acid biosynthesis and metabolism |
| **PP_1830** | *aroC* | PA14_42760 | PA1681 | Amino acid biosynthesis and metabolism |
| **PP_1917** | *pabC* | PA14_25710 | PA2964 | Amino acid biosynthesis and metabolism |
| **PP_2329** | *pabB* | PA14_41820 | PA1758 | Amino acid biosynthesis and metabolism |
| **PP_5078** | *aroB* | PA14_66600 | PA5038 | Amino acid biosynthesis and metabolism |
| **PP_0528** | *ispA* | PA14_11560 | PA4569 | Biosynthesis of cofactors, prosthetic groups and carriers |
| **PP_0602** | *ribF* | PA14_60380 | PA4561 | Biosynthesis of cofactors, prosthetic groups and carriers |
| **PP_0787** | *nadC* | PA14_58700 | PA4524 | Biosynthesis of cofactors, prosthetic groups and carriers |
| **PP_1231** | *nadA* | PA14_51330 | PA1004 | Biosynthesis of cofactors, prosthetic groups and carriers |
| **PP_1426** | *nadB* | PA14_54450 | PA0761 | Biosynthesis of cofactors, prosthetic groups and carriers |
| **PP_1997** | *folC* | PA14_23880 | PA3111 | Biosynthesis of cofactors, prosthetic groups and carriers |
| **PP_3999** | *cysG* | PA14_30340 | PA2611 | Biosynthesis of cofactors, prosthetic groups and carriers |
| **PP_4784** | *hemL* | PA14_12390 | PA3977 | Biosynthesis of cofactors, prosthetic groups and carriers |
| **PP_1777** | *xanA* | PA14_70270 | PA5322 | Carbohydrate transport and metabolism |
| **PP_2012** | *nadK* | PA14_24220 | PA3038 | Carbohydrate transport and metabolism |
| **PP_3821** | *galU* | PA14_38350 | PA2023 | Carbohydrate transport and metabolism |
| **PP_4965** | *tktA* | PA14_07130 | PA0548 | Carbohydrate transport and metabolism |
| **PP_0964** | *murA* | PA14_57810 | PA4450 | Cell envelope biogenesis, outer membrane |
| **PP_1335** | *murD* | PA14_57360 | PA4413 | Cell envelope biogenesis, outer membrane |
| **PP_2862** | *bacA* | PA14_39190 | PA1959 | Cell envelope biogenesis, outer membrane |
| **PP_4716** | *glmM* | PA14_39190 | PA1959 | Cell envelope biogenesis, outer membrane |
| **PP_5411** | *glmU* | PA14_73220 | PA5552 | Central intermediary metabolism |
| **PP_0520** | *pgpA* | PA14_11470 | PA4051 | Fatty acid and phospholipid metabolism |
| **PP_0565** | *idi* | PA14_64010 | PA4841 | Fatty acid and phospholipid metabolism |
| **PP_1520** | *plsB* | PA14_16860 | PA3673 | Fatty acid and phospholipid metabolism |
| **PP_1614** | *ispD* | PA14_17340 | PA3633 | Fatty acid and phospholipid metabolism |
| **PP_1618** | *ispF* | PA14_17420 | PA3627 | Fatty acid and phospholipid metabolism |
| **PP_1844** | *plsC* | PA14_00060 | PA0005 | Fatty acid and phospholipid metabolism |
| **PP_1913** | *fabD* | PA14_25650 | PA2968 | Fatty acid and phospholipid metabolism |
| **PP_2734** | *cfa* | PA14_73140 | PA5546 | Fatty acid and phospholipid metabolism |
| **PP_4175** | *fabB* | PA14_43690 | PA1609 | Fatty acid and phospholipid metabolism |
| **PP_4935** | *msbA* | PA14_66080 | PA4997 | Fatty acid and phospholipid metabolism |
| **PP_5364** | *cls* | PA14_71130 | PA5394 | Fatty acid and phospholipid metabolism |
| **PP_0565** | *-* | PA14_64010 | PA0565 | Fatty acid and phospholipid metabolism |
| **PP_0849** | *ndk* | PA14_14820 | PA3807 | Nucleotide biosynthesis and metabolism |
| **PP_1032** | *guaA* | PA14_1534 | PA3769 | Nucleotide biosynthesis and metabolism |
| **PP_3989** | *dcm* | _ | _ | Nucleotide biosynthesis and metabolism |
| **PP_4810** | *nadD* | PA14_12020 | PA4006 | Nucleotide biosynthesis and metabolism |
| **PP_4869** | *nadE* | PA14_64980 | PA4920 | Nucleotide biosynthesis and metabolism |
| **PP_4889** | *purA* | PA14_65230 | PA4938 | Nucleotide biosynthesis and metabolism |
| **PP_5296** | *gmk-2* | PA14_70440 | PA5336 | Nucleotide biosynthesis and metabolism |
