# Supplementary material for: A genome-scale metabolic reconstruction of Pseudomonas putida KT2440: iJN746 as a cell factory
Source: BMC Syst Biol. 2008 Sep 16;2:79. doi: 10.1186/1752-0509-2-79 (PMC2569920; doi:10.1186/1752-0509-2-79)
Supplement: Additional file 5 — Table S4. False-negative essential genes in iJN746. Genes that were not predicted to be essential in iJN746 but were reported as essential genes in P. aeruginosa [83]. [file 1752-0509-2-79-S5.doc]

**Additional file 5: Table S4.** False-negative essential genes in *i*JN746. Genes that were not predicted to be essential in *i*JN746 but were reported as essential genes in *P. aeruginosa* [83]*.* Word file.

| **P. putida**  **gene** | **gene** | **PA14**  **orthologo** | **PA01**  **orthologo** | **Functional Class** |
| --- | --- | --- | --- | --- |
| **#PP_0060** | glyS | PA14_00090 | PA0008 | Amino acid biosynthesis and metabolism |
| **#PP_0061** | glyQ | PA14_00100 | PA0009 | Amino acid biosynthesis and metabolism |
| **#PP_0067** | fmt | PA14_00190 | PA0018 | Translation, ribosomal structure and biogenesis |
| **#PP_0603** | ileS | PA14_60370 | PA4560 | Amino acid biosynthesis and metabolism |
| **#PP_0930** | gatB | PA14_58190 | PA4484 | Amino acid biosynthesis and metabolism |
| **#PP_0932** | gatC | PA14_58170 | PA4482 | Amino acid biosynthesis and metabolism |
| **#PP_1205** | proS | PA14_51900 | PA0956 | Amino acid biosynthesis and metabolism |
| **#PP_1496** | lysS | PA14_16530 | PA3700 | Amino acid biosynthesis and metabolism |
| **#PP_2469** | pheS | PA14_28690 | PA2740 | Amino acid biosynthesis and metabolism |
| **#PP_2904** | glnS | PA14_41380 | PA1794 | Amino acid biosynthesis and metabolism |
| **#PP_2905** | cysS | PA14_41360 | PA1795 | Amino acid biosynthesis and metabolism |
| **#PP_4000** | serS | PA14_30330 | PA2612 | Amino acid biosynthesis and metabolism |
| **#PP_5046** | glnA | PA14_67600 | PA5119 | Amino acid biosynthesis and metabolism |
| **$PP_0415** | rpe | PA14_07910 | PA0607 | Carbohydrate transport and metabolism |
| **$PP_4188** | kgdB | PA14_44000 | PA1586 | Central intermediary metabolism |
| **$PP_4189** | kgdA | PA14_44010 | PA1585 | Central intermediary metabolism |
| **$PP_4190** | sdhB | PA14_44020 | PA1584 | Central intermediary metabolism |
| **$PP_4191** | sdhA | PA14_44030 | PA1583 | Central intermediary metabolism |
| **$PP_4192** | sdhD | PA14_44050 | PA1582 | Central intermediary metabolism |
| **$PP_4193** | sdhC | PA14_44060 | PA1581 | Central intermediary metabolism |
| **$PP_5150** | rpiA | PA14_04310 | PA0330 | Carbohydrate transport and metabolism |
| **$PP_5415** | atpA | PA14_73260 | PA5556 | Energy production and conversion |
| **$PP_5418** | atpE | PA14_73300 | PA5559 | Energy production and conversion |
| ***PP_0322,(PP_0671)** | glyA-1,  (glyA-2) | PA14_60890 | PA4602 | Amino acid biosynthesis and metabolism |
| ***PP_1593,(PP1771)** | pyrH,(cmk) | PA14_17080 | PA3654 | Nucleotide biosynthesis and metabolism |
| ***PP_1602,( PP_4174)** | fabZ,(fabA) | PA14_17190 | PA3645 | Fatty acid and phospholipid metabolism |
| ***PP_1945, (PP_2265)** | folD-1,  (folD-2) | PA14_41350 | PA1796 | Biosynthesis of cofactors, prosthetic groups, and carriers |
| **PP_0073** | hemF | PA14_00280 | PA0024 | Biosynthesis of cofactors, prosthetic groups, and carriers |
| **PP_0514** | ribD | PA14_11400 | PA4056 | Nucleotide biosynthesis and metabolism |
| **PP_0522** | ribA | PA14_11510 | PA4047 | Nucleotide biosynthesis and metabolism |
| **PP_0736** | murI | PA14_61660 | PA4662 | Cell envelope biogenesis, outer membrane |
| **PP_0849** | ndk | PA14_14820 | PA3807 | Nucleotide biosynthesis and metabolism |
| **PP_1129** | pdxH | PA14_50800 | PA1049 | Nucleotide biosynthesis and metabolism |
| **PP_1989** | asd | PA14_23800 | PA3117 | Amino acid biosynthesis and metabolism |
| **PP_4725** | dapB | PA14_62940 | PA4759 | Amino acid biosynthesis and metabolism |
| **PP_4960** | fda | PA14_07230 | PA0555 | Carbohydrate transport and metabolism |
| **PP_5040** | fbp | PA14_67490 | PA5110 | Carbohydrate transport and metabolism |

**Table S4.** False-negative essentials genes in *i*JN746. Genes which were not predicted to be essential in *i*JN746 but were reported as essential genes in *P. aeruginosa.* (♯) genes which encode for tRNA synthetases, ($) genes which mutation result in a non lethal phenotype, however, present a significant effect on the growth rate, (*) couple of gene which encode for isoenzymes in *Pseudomonas*.
