# Supplementary material for: A genome-scale metabolic reconstruction of Pseudomonas putida KT2440: iJN746 as a cell factory
Source: BMC Syst Biol. 2008 Sep 16;2:79. doi: 10.1186/1752-0509-2-79 (PMC2569920; doi:10.1186/1752-0509-2-79)
Supplement: Additional file 6 — Table S5. Predicted essential genes in Glucose-iM9 minimal medium. Not shown are genes that were also predicted to be essential in iLB rich medium. [file 1752-0509-2-79-S6.doc]

**Additional file 6: Table S5.** Predicted essential genes in Glucose-*i*M9 minimal medium. Not shown are genes that were also predicted to be essential in *i*LB rich medium. Word file.

| **KT2440**  **gene** | **Gene name** | **Funcional Class** |
| --- | --- | --- |
| **PP_0082** | *trpA* | Amino acid biosynthesis and metabolism |
| **PP_0083** | *trpB* | Amino acid biosynthesis and metabolism |
| **PP_0184** | *argH* | Amino acid biosynthesis and metabolism |
| **PP_0289** | *hisB* | Amino acid biosynthesis and metabolism |
| **PP_0290** | *hisH* | Amino acid biosynthesis and metabolism |
| **PP_0292** | *hisA* | Amino acid biosynthesis and metabolism |
| **PP_0293** | *hisF* | Amino acid biosynthesis and metabolism |
| **PP_0417** | *trpE* | Amino acid biosynthesis and metabolism |
| **PP_0420** | *trpG* | Amino acid biosynthesis and metabolism |
| **PP_0421** | *trpD* | Amino acid biosynthesis and metabolism |
| **PP_0422** | *trpC* | Amino acid biosynthesis and metabolism |
| **PP_0515** | *ridE* | Nucleotide biosynthesis and metabolism |
| **PP_0517** | *ridH* | Nucleotide biosynthesis and metabolism |
| **PP_0614** | *-* | Amino acid biosynthesis and metabolism |
| **PP_0840** | *cysE* | Nucleotide biosynthesis and metabolism |
| **PP_0860** | *piuB-cysJ* | Nucleotide biosynthesis and metabolism |
| **PP_0965** | *hisG* | Amino acid biosynthesis and metabolism |
| **PP_0966** | *hisD* | Nucleotide biosynthesis and metabolism |
| **PP_0967** | *hisC* | Nucleotide biosynthesis and metabolism |
| **PP_1025** | *leuA* | Nucleotide biosynthesis and metabolism |
| **PP_1086** | *pyrC* | Nucleotide biosynthesis and metabolism |
| **PP_1088** | *argG* | Amino acid biosynthesis and metabolism |
| **PP_1303** | *cysD* | Nucleotide biosynthesis and metabolism |
| **PP_1304** | *cysN* | Nucleotide biosynthesis and metabolism |
| **PP_1525** | *dapE* | Nucleotide biosynthesis and metabolism |
| **PP_1620** | *surE* | Cellular processes |
| **PP_1769** | *pheA* | Nucleotide biosynthesis and metabolism |
| **PP_1815** | *pyrF* | Nucleotide biosynthesis and metabolism |
| **PP_1985** | *leuC* | Amino acid biosynthesis and metabolism |
| **PP_1986** | *leuD* | Nucleotide biosynthesis and metabolism |
| **PP_1988** | *leuB* | Nucleotide biosynthesis and metabolism |
| **PP_1995** | *trpF* | Nucleotide biosynthesis and metabolism |
| **PP_2095** | *pyrD* | Nucleotide biosynthesis and metabolism |
| **PP_2328** | *cysH* | Amino acid biosynthesis and metabolism |
| **PP_2371** | *cysI* | Amino acid biosynthesis and metabolism |
| **PP_3511** | *ilvE* | Amino acid biosynthesis and metabolism |
| **PP_3633** | *argC* | Amino acid biosynthesis and metabolism |
| **PP_3721** | *aspC/aruH* | Amino acid biosynthesis and metabolism |
| **PP_4038** | *-* | Nucleotide biosynthesis and metabolism |
| **PP_4481** | *aruC/argD* | Amino acid biosynthesis and metabolism |
| **PP_4565** | *aceK* | Amino acid biosynthesis and metabolism |
| **PP_4678** | *ilvC* | Amino acid biosynthesis and metabolism |
| **PP_4679** | *ilvN* | Amino acid biosynthesis and metabolism |
| **PP_4680** | *ilvB* | Amino acid biosynthesis and metabolism |
| **PP_4699** | *panB* | Biosynthesis of cofactors, prosthetic groups, and carriers |
| **PP_4700** | *panC* | Biosynthesis of cofactors, prosthetic groups, and carriers |
| **PP_4725** | *dapB* | Amino acid biosynthesis and metabolism |
| **PP_4822** | *purH* | Nucleotide biosynthesis and metabolism |
| **PP_4998** | *pyrB* | Nucleotide biosynthesis and metabolism |
| **PP_5014** | *hisI* | Amino acid biosynthesis and metabolism |
| **PP_5015** | *hisE* | Amino acid biosynthesis and metabolism |
| **PP_5128** | *ilvD* | Amino acid biosynthesis and metabolism |
| **PP_5289** | *argB* | Amino acid biosynthesis and metabolism |
| **PP_5291** | *pyrE* | Nucleotide biosynthesis and metabolism |
