# Supplementary material for: A genome-scale metabolic reconstruction of Pseudomonas putida KT2440: iJN746 as a cell factory
Source: BMC Syst Biol. 2008 Sep 16;2:79. doi: 10.1186/1752-0509-2-79 (PMC2569920; doi:10.1186/1752-0509-2-79)
Supplement: Additional file 7 — Table S6. PHA polymer composition found in different Pseudomonas strains sorted by carbon sources. [file 1752-0509-2-79-S7.doc]

**Additional file 7: Table S6.** PHA polymer composition found in different *Pseudomonas* strains sorted by carbon sources. Word file.

| **Strain** | **Carbon source** | **PHA %**  **(Wt/Wt)** | **Polymer composition**  **(%)** | | | | | | | **Reference** |
| --- | --- | --- | --- | --- | --- | --- | --- | --- | --- | --- |
|  |  |  | **C6** | **C8** | **C10** | **C12** | **C12:1** | **C14** | **C14:1** |  |
| **Pseudomonas sp. Strain NCIMB 40135** | Acetate | 5 |  |  |  |  |  |  |  | [102] |
|  | Glycerol | 5 |  |  |  |  |  |  |  | [102] |
|  | Lactate | 9 |  |  |  |  |  |  |  | [102] |
|  | Succinate | 1.3 |  |  |  |  |  |  |  | [102] |
|  | Glucose | 8 |  |  |  |  |  |  |  | [102] |
|  | Gluconate | 17 |  |  |  |  |  |  |  | [102] |
|  | Fructose | 16 |  |  |  |  |  |  |  | [102] |
|  | Octanoate | 66 |  |  |  |  |  |  |  | [102] |
| **P. putida DSM 291** | Gluconate | 28.8 | 5.9 | 34.4 | 45.2 | 18.4 |  |  |  | [85] |
|  | 0ctanoate | 40.3 | 2.6 | 89.6 | 5.4 | 2.4 |  |  |  | [85] |
| **P.putida KT2440/2** | Octanoate | 47.1 | 8 | 91 | 1 | ND |  |  |  | [103] |
|  | Glucose | 16.9 | Traces | 6.9 | 74.3 | 7 | 8.8 | Traces | 1.6 | [17] |
|  | Fructose | 24.5 | 0.5 | 12.6 | 70.8 | 5.7 | 8.5 | 0.3 | 1.6 | [17] |
|  | Glycerol | 22.0 | 1.7 | 21.4 | 63.6 | 3.8 | 8.6 | 0.1 | 0.8 | [17] |
|  | Decanoate | 27.6 | 5.3 | 52.3 | 42.3 | ND | ND | ND | ND | [17] |
|  | Hexanoate |  | 72 | 14 | 10 | 1 | 1 | 1 | 1 | [61] |
| **P. putida CA-3** | Phenylacetic | 30 |  |  |  |  |  |  |  | [84] |

C6, 3-hydroxyhexanoate; C8, 3-hydroxyoctanoate; C10, 3-hydroxydecanoate; C12:1, 3-hydroxy-5-cis-dodecenoate; C12, 3-hydroxydodecanoate; C14:1, 3-hydroxy-7-cis-tetradecenoate; C14, 3-hydroxytetradecanoate. ND, not detected.
