# Supplementary material for: A genome-scale metabolic reconstruction of Pseudomonas putida KT2440: iJN746 as a cell factory
Source: BMC Syst Biol. 2008 Sep 16;2:79. doi: 10.1186/1752-0509-2-79 (PMC2569920; doi:10.1186/1752-0509-2-79)
Supplement: Additional file 10 — Table S9. List of biomass components in iJN746. This file contains the complete list of compounds which are part of Pseudomonas putida biomass. [file 1752-0509-2-79-S10.doc]

**Additional file 10: Table S9.** List of biomass components in *i*JN746. This file contains the complete list of compounds which are part of *Pseudomonas* *putida* biomass. Word file.

| **Abbreviation** | **mmol/gDW** | **Abbreviation** | **mmol/gDW** |
| --- | --- | --- | --- |
| **5mthf** | 0.05 | **leu-L** | 0.326 |
| **accoa** | 0.00005 | **lys-L** | 0.146 |
| **ala-L** | 0.488 | **met-L** | 0.00215 |
| **amp** | 0.001 | **nad** | 0.00005 |
| **arg-L** | 0.281 | **nadh** | 0.00013 |
| **asn-L** | 0.229 | **nadp** | 0.0004 |
| **asp-L** | 0.229 | **nadph** | 0.0005 |
| **atp** | 457.318 | **pe120** | 0.0005 |
| **clpn120** | 0.0005 | **pe160** | 0.0005 |
| **clpn160** | 0.0005 | **pe161** | 0.0005 |
| **clpn161** | 0.0005 | **pe180** | 0.0005 |
| **clpn180** | 0.0005 | **pe181** | 0.0005 |
| **clpn181** | 0.0005 | **peptido_kt** | 0.028 |
| **coa** | 0.000006 | **pg120** | 0.0005 |
| **cpe160** | 0.0005 | **pg160** | 0.0005 |
| **cpe180** | 0.0005 | **pg180** | 0.0005 |
| **cpg160** | 0.0005 | **phe-L** | 0.176 |
| **cpg180** | 0.0005 | **pro-L** | 0.035 |
| **ctp** | 0.126 | **ptrc** | 0.035 |
| **cys-L** | 0.087 | **ser-L** | 0.205 |
| **datp** | 0.0247 | **sheme** | 0.0005 |
| **dctp** | 0.0254 | **succoa** | 0.000003 |
| **dgtp** | 0.0247 | **thr-L** | 0.241 |
| **fad** | 0.00001 | **trp-L** | 0.054 |
| **gln-L** | 0.25 | **tyr-L** | 0.131 |
| **glu-L** | 0.25 | **udpg** | 0.003 |
| **gly** | 0.203 | **utp** | 0.136 |
| **gtp** | 455.608 | **val-L** | 0.402 |
| **h2o** | 0.0005 | **adp** | 455.608 |
| **hemeO** | 0.09 | **h** | 4.556.035 |
| **his-L** | 0.276 | **pi** | 455.628 |
| **ile-L** | 0.428 | **ppi** | 0.7302 |
